# CHCHD4 Oxidoreductase Activity: A Comprehensive Analysis of the Molecular, Functional, and Structural Properties of Its Redox-Regulated Substrates

**DOI:** 10.3390/molecules30102117

**Published:** 2025-05-10

**Authors:** Nicole Balasco, Nazanine Modjtahedi, Alessandra Monti, Menotti Ruvo, Luigi Vitagliano, Nunzianna Doti

**Affiliations:** 1 Institute of Molecular Biology and Pathology, National Research Council (CNR), Department of Chemistry, University of Rome Sapienza, Piazzale Aldo Moro 5, 00185 Rome, Italy; nicole.balasco@cnr.it; 2 Unité Physiopathologie et Génétique du Neurone et du Muscle, UMR CNRS 5261, Inserm U1315, Université Claude Bernard Lyon 1, 69008 Lyon, France; nazanine.modjtahedi@univ-lyon1.fr; 3 Institute of Biostructures and Bioimaging, National Research Council (CNR), Via P. Castellino 111, 80131 Naples, Italy; alessandra.monti@ibb.cnr.it (A.M.); menotti.ruvo@cnr.it (M.R.)

**Keywords:** protein-protein interactions, protein structure prediction, p53

## Abstract

The human CHCHD4 protein, which is a prototypical family member, carries a coiled–coil–helix–coiled–coil–helix motif that is stabilized by two disulfide bonds. Using its CPC sequence motif, CHCHD4 plays a key role in mitochondrial metabolism, cell survival, and response to stress conditions, controlling the mitochondrial import of diversified protein substrates that are specifically recognized through an interplay between covalent and non-covalent interactions. In the present review, we provide an updated and comprehensive analysis of CHCHD4 substrates controlled by its redox activities. A particular emphasis has been placed on the molecular and structural aspects of these partnerships. The literature survey has been integrated with the mining of structural databases reporting either experimental structures (Protein Data Bank) or structures predicted by AlphaFold, which provide protein three-dimensional models using machine learning-based approaches. In providing an updated view of the thirty-four CHCHD4 substrates that have been experimentally validated, our analyses highlight the notion that this protein can operate on a variety of structurally diversified substrates. Although in most cases, CHCHD4 plays a crucial role in the formation of disulfide bridges that stabilize helix–coil–helix motifs of its substrates, significant variations on this common theme are observed, especially for substrates that have been more recently identified.

## 1. Introduction

Protein–protein interactions (PPIs) represent key and ubiquitous regulatory events in all biological processes, since these macromolecules generally use their three-dimensional structures, which can be characterized by a wide range of different flexibility, to interact with each other and with other biomolecules. The modulation of PPI is, therefore, a powerful mechanism for the up- or down-regulation of key biochemical pathways in both physiological and pathological contexts. A detailed understanding of the chemical and structural basis of these interactions is paramount to deciphering intricate cellular mechanisms and developing targeted therapeutic interventions [1,2,3,4,5,6,7,8]. However, the chemical complexity of proteins, combined with their considerable intrinsic flexibility, makes the understanding of the structural basis of their partnerships a cumbersome and sometimes puzzling task that is the subject of intense research activities [6]. The large variability of the physico-chemical properties of the amino acid residues that constitute proteins leads to protein–protein interfaces whose stabilization relies on many different intermolecular forces.

In many biological processes, the formation of protein–protein complexes relies on the concomitant formation of covalent bonds, which often involves the oxidation of cysteine residues as well as non-covalent interactions. The synergy of these interactions is fundamental for the correct formation of the covalent linkage. Indeed, considering the abundance of cysteine residues on protein surfaces, the contribution of non-covalent interactions is fundamental for the formation of specific disulfide bridges under oxidizing conditions [9]. Although the action of redox catalysts and disulfide reshuffling are common processes used to correct mispaired and potentially harmful non-physiological disulfide bridges, the occurrence of specific non-covalent interactions is a further major factor in determining the functional exact pairing of cysteine residues.

The interaction of the human oxidoreductase CHCHD4 (hereafter hCHCHD4) with its many substrates represents an illuminating example of the interplay between covalent and non-covalent interactions in the protein–protein recognition process. This mitochondrial protein, which is a member of the coiled–coil–helix–coiled–coil–helix domain (CHCHD) family, is the focus of multiple research activities in cell biology and biochemistry, particularly those related to mitochondrial metabolism, cell survival, and cellular responses to oxidative stress [10,11]. Since CHCHD4 and its protein substrates are emerging as a new class of proteins associated with various pathologies, it is of particular importance to define the role of the CHCHD4-dependent mitochondrial import pathway in health and disease, including mitochondrial disorders, neurodegeneration, and cancer [10,12,13,14,15,16,17,18,19,20,21,22,23,24,25,26,27,28,29,30,31,32,33,34,35,36,37,38].

In the present review, we comprehensively describe the functional, molecular, and structural properties of the regulated substrates. The evolutionarily conserved hCHCHD4 protein and its putative substrates have been the subject of very interesting literature reviews [10,11,37,39,40,41,42], but these have essentially focused on the functional and pathological aspects of the redox-regulated substrates. Here, after presenting a detailed description of the notations used in this manuscript, along with an overview of the literature search methodology and the procedures employed to predict the three-dimensional structures of the hCHCHD4 redox ligands (Section 2), we offer a concise summary of the fundamental molecular and functional properties of this protein, as well as its extensively studied yeast homolog, yMIA40 (see Section 3). Then, we focus on the molecular players in the interaction and recognition of the substrates by the hCHCHD4 oxidase, with special attention paid to the Cys residues involved in the formation of a crucially transient disulfide bridge (Section 4). Furthermore, we inspect the Protein Data Bank (PDB) [43] for the three-dimensional structure of hCHCHD4 and its complexes (Section 5). However, since the available experimental structural data are extremely limited, in Section 6, we have integrated this information by searching the EBI-AlphaFold database [44] and performing exhaustive predictions of the three-dimensional structures of CHCHD4 redox-regulated substrates using the innovative and powerful approach based on machine-learning implemented in AlphaFold (AF), which provides accurate atomic-level information on proteins [45,46].

## 2. Methodology

In the literature, the human protein CHCHD4 has often been identified using multiple notations. Sometimes, the acronyms CHCHD4 and MIA40 have been used as synonyms. To avoid confusion, here we have used the term hCHCHD4 to identify the human protein. For other mammalian orthologs, CHCHD4 has been preceded by a symbol for the species (for example, the *Mus musculus* ortholog has been denoted as mCHCHD4). Mia40 has been exclusively used to denote the *S. cerevisiae* ortholog (yMia40). For human proteins, we have used acronyms with all capital letters, whereas for yeast proteins, only the first letter of the acronym has been reported in uppercase.

### 2.1. Survey of Literature

In this study, we conducted a comprehensive analysis of articles identifying and describing the redox-regulated substrates of hCHCHD4, independently of the presence of previously defined canonical or non-canonical motifs [10,17]. Our literature search was based on multiple queries on PUBMED and Google Scholar, updated until February 2025. Given that hCHCHD4 is also referred to as human MIA40, we performed several distinct searches on PUBMED (Table 1).

Following this initial survey, which identified 150 manuscripts of potential interest, we conducted an additional survey using Google Scholar, which consisted of the inspection of all papers citing two seminal works in the field, i.e., Modjtahedi et al. [11] and Banci et al. [47]. This further search led to the identification of 57 additional manuscripts.

All selected articles were individually reviewed by selecting reports on the topic. The redox-regulated substrates identified are reported in Table 2 in Section 4.

### 2.2. Survey of the Protein Data Bank

The search for data related to the three-dimensional structure of hCHCHD4 and its complexes has been conducted by interrogating the Blast server (https://blast.ncbi.nlm.nih.gov/, accessed on 1 March 2025) using the sequence of the protein as the query and the PDB as the database to be searched. As detailed below, only two entries have been identified. To increase the amount of structural information, we included in our searches the sequences of CHCHD4 orthologs. In this second search, we have identified three additional structures (refer to Table 3 in Section 5). It is worth mentioning that some structural information has been reported for the complexes of the protein with COX17, ALR, TIM9, and TIM10 [47,48,49,50].

### 2.3. AlphaFold Predictions

In addition to the data retrieved from the PDB, models of substrates have been predicted with the AlphaFold3 (AF3) algorithm (https://alphafoldserver.com/, accessed on 1 March 2025) using default settings [46] and compared with those reported in the AlphaFold database at EMBL-EBI (https://alphafold.ebi.ac.uk/, accessed on 1 March 2025). The best-predicted model (model 0) out of the five provided by AF3 was considered. The reliability of these predictions has been assessed by analyzing the Predicted Aligned Error (PAE) matrices and the per-residue Local Distance Difference Test (pLDDT) [51] for each predicted structure.

Adopting the approach used in the experimental studies [49], in the prediction of the hCHCHD4-substrate complexes, to avoid the formation of non-physiological disulfide bridges, the sequence of the proteins has been slightly modified. More specifically, for hCHCHD4, by mutating Cys53 of the Cys53-Pro54-Cys55 (CPC) motif to Ser, only the reactive Cys55 was kept in its reduced state and ready to react. For the prediction of individual substrates, the analysis of their intrinsic structural properties was conducted by using the wild-type sequence in the prediction.

## 3. The hCHCHD4/yMia40 World

As anticipated in the previous sections, the focus of this review is the description of the functional, molecular, and structural properties of the hCHCHD4 redox-regulated substrates. In the following paragraphs, we also provide a brief description of the basic molecular and functional properties of this protein needed to provide the reader with the essential information necessary to contextualize the rest of the paper. Since in the literature, there has been a frequent sharing of information between hCHCHD4 and yMia40, some data directly related to this latter protein will also be described.

### 3.1. Evolution and Molecular Organization of hCHCHD4/yMia40

hCHCHD4 is the core component of a redox-regulated mitochondrial import machinery that is located in the mitochondrial intermembrane space (IMS) [11,52]. As mentioned above, along with some significant differences, it shares several molecular and functional features with its highly investigated yeast homolog (yMia40) despite the evolutionary distance between the two organisms, *H. sapiens* versus *S. cerevisiae* [53,54,55]. Although hCHCHD4 is significantly smaller (about 16 kDa) than yMia40 (about 40 kDa), since the former lacks the N-terminal segment found in the latter, these two proteins exhibit sequence similarity reaching 75% in the aligned regions (residues 1–142 and 248–286 for hCHCHD4 and yMia40, respectively) that contain the highly conserved redox-active CPC motif and the structural twin cysteine motif CX_9_C-CX_9_C [11,47,53,56,57,58,59].

The N-terminal segment present exclusively on yMia40 carries a mitochondrial targeting signal and a transmembrane region that enables the protein to anchor to the inner mitochondrial membrane (IMM) [34,35,36,37,38,39]. On the other hand, hCHCHD4, which lacks the mitochondrial targeting signal, is consequently fully soluble in the IMS. Notably, hCHCHD4 gets imported and localized to the IMM via its interaction with a mitochondrial protein called apoptosis-inducing factor (AIF) [19]. AIF is a phylogenetically conserved flavoprotein anchored to the IMM that was initially identified as a caspase-independent effector of cell death [60,61]. Upon several apoptotic stimuli and the permeabilization of the mitochondrial outer membrane (MOMP), AIF is released from the mitochondria and translocates to the nucleus, where it facilitates chromatin condensation and DNA degradation, through its interaction with the cytosolic protein CypA [20,60,62,63,64]. In its physiological location, AIF is now recognized as playing key regulatory roles in the mitochondrial import of hCHCHD4 and the optimal functioning of the hCHCHD4-dependent protein import pathway, which, among other things, controls mitochondrial biogenesis and function [17,19,23,32,65,66,67,68,69]. In humans, the importance of AIF/hCHCHD4 interaction is underscored by the observation that mitochondrial disease-associated pathogenic mutations of AIF impair its binding to hCHCHD4 and/or impact hCHCHD4 expression levels [17,19,23,32].

### 3.2. The Mitochondrial Functions of hCHCHD4/yMia40 and Their Mechanism of Action

The primary biological function of hCHCHD4 and yMia40 in the IMS is to oxidize cysteine side-chain thiols in incoming mitochondrial substrate proteins, leading to the formation of vital intramolecular disulfide bonds that are crucial for proper protein folding, stability, and retention in the mitochondrion [16,35,47,53,56,70,71,72,73,74,75,76,77,78,79]. The mechanism of hCHCHD4-dependent oxidative protein folding is well documented at the molecular level, but the structural basis has only been elucidated for a small number of substrates (see below).

Briefly, the redox-regulated protein import machinery involving these proteins, known as the mitochondrial disulfide relay system (DRS) or disulfide relay-dependent MIA (Mitochondrial Import and Assembly) import pathway, was initially discovered in the yeast *S. cerevisiae* [52,56,57,59,80,81,82,83,84,85,86,87,88,89]. Over the years, it has been demonstrated that in addition to their redox-active cysteines, substrates of yMia40 contain intermembrane space-targeting signals (ITS), also referred to as mitochondrial intermembrane space-sorting signals (MISS) [48,90]. The main features of the ITS motifs are as follows: (i) their presence either upstream or downstream of the cysteine targeted by yMia40; (ii) they are necessary and sufficient to cross the outer mitochondrial membrane; (iii) they form an amphipathic helix with hydrophobic residues facing the docking cysteine side and dispensable charged residues on the other side, and (iv) they fit into the substrate-binding cleft of yMia40 via hydrophobic interactions [39,48,90,91,92,93]. Following an initial noncovalent interaction of the ITS with the hydrophobic groove of yMia40, which is adjacent to the CPC motif, the substrate protein forms a transient intermolecular disulfide bond with the substrate-binding domain of yMia40 [48]. The subsequent oxidation of the substrate proteins leads to the formation of intramolecular disulfide bonds, promoting proper protein folding and effectively trapping them in the IMS. Finally, yMia40 undergoes reoxidation through electron transfer mediated by the sulfhydryl oxi dase Erv1, via its N-terminal CXnC non-canonical motif (Cys30/Cys33). These electrons move to the FAD-proximal CX2C pair (Cys130/Cys133), then to the flavin moiety and to cytochrome c, cytochrome oxidase (Cox), and finally to molecular oxygen. Alternatively, the electrons released by Erv1 can be captured by cytochrome c peroxidase (Ccp1) [94,95,96]. While it is established that this pathway can function under anaerobic conditions, the identity of the final electron acceptor in such a scenario remains unclear [82,97,98]. In human cells, the reoxidation of the CPC motif of hCHCHD4 is ensured by ALR (Augmenter of Liver Regeneration), the homolog of the *S. cerevisiae* Erv1 enzyme [70,72].

In addition, hCHCHD4, like yMia40, plays an important role in the mitochondrial iron–sulfur export (ISE) machinery as it is involved in the export of iron–sulfur clusters from mitochondria, the maturation of cytosolic iron–sulfur clusters, and cellular iron homeostasis [99,100,101]. Iron–sulfur clusters are prosthetic groups required for several biological activities, including electron transfer, DNA repair, regulation of gene expression, response to oxidative stress, regulatory processes, and iron–sulfur sources [102]. Thus, alterations in the iron–sulfur cluster biogenesis pathway are related to human diseases including Friedreich’s ataxia, sideroblastic anemia, and hereditary skeletal muscle disease [102].

### 3.3. Involvement of hCHCHD4 in Pathological States

Given the centrality of the MIA pathway in mitochondrial physiology, the dysregulation or malfunction of hCHCHD4 or its regulatory partners AIF and ALR have been implicated in various pathological states [10,17,103,104,105]. For example, changes in hCHCHD4 expression levels have been linked to mitochondrial disorders that can lead to severe systemic manifestations [17]. Knowing how critical the impact of redox regulation and signaling is to the optimal and finely tuned functioning of the MIA pathway, the role of hCHCHD4 and its regulatory partners in response to oxidative stress positions them as noteworthy players in neurodegenerative diseases, where mitochondrial dysfunction is a common event [17,52,79]. The hCHCHD4 pathway has also been implicated in cancer biology, particularly in the context of tumor metabolism, the response to hypoxia, and cell survival under stress conditions [13,106]. Aberrant expression levels of hCHCHD4 can influence cellular redox states and contribute to the malignant phenotype by allowing tumor cells to thrive in environments with high oxidative stress [18,107]. Very recent reviews discussing the role of hCHCHD4 in the IMS, either in terms of physiological or pathological implications, have been published [10,17,35,52].

## 4. Redox Substrates of hCHCHD4/yMIA40

Previous studies, essentially realized in yeast, indicated that the prototypical nuclear-encoded redox-regulated substrates of the MIA or DRS pathway are small proteins, typically less than 25 kDa in size. The structure of these substrates is characterized by the presence of two α-helices, each bearing a pair of cysteine residues that are separated by either three or nine amino acid residues. Based on this feature, substrates were denoted either as twin CX_3_C or twin CX_9_C proteins [52,108,109]. Twin CX_9_C proteins present a characteristic coiled–coil–helix–coiled–coil–helix (CHCH) structural fold, which is stabilized, in their oxidized forms, by two disulfide bonds. Despite this common structural feature, these proteins exhibit remarkable variations at the primary sequence level that have somehow complicated the accurate prediction of their structural models [110]. The implication of these proteins in various physio-pathological contexts has been highlighted in various reviews [10,11,17,35,108,109,111]. For example, Modjtahedi and colleagues (2016) explored the role of CX_9_C motif-containing proteins in mitochondrial respiratory complexes’ biogenesis and their potential as therapeutic targets for human diseases, and showed the critical role of the hCHCHD4/yMIA40 import pathway in cell survival [11].

Most of the known substrates of hCHCHD4 belong to the twin CX_9_C protein family [11,17,109,112]. However, as also emerged from our updated survey, an increasing number of redox-substrate proteins lacking canonical motifs have been identified, thus showing the great versatility of hCHCHD4 and yMia40 in processing proteins endowed with diverse structures [70,101,113,114,115,116,117,118,119,120,121,122,123,124,125,126,127,128].

**Figure 1:**
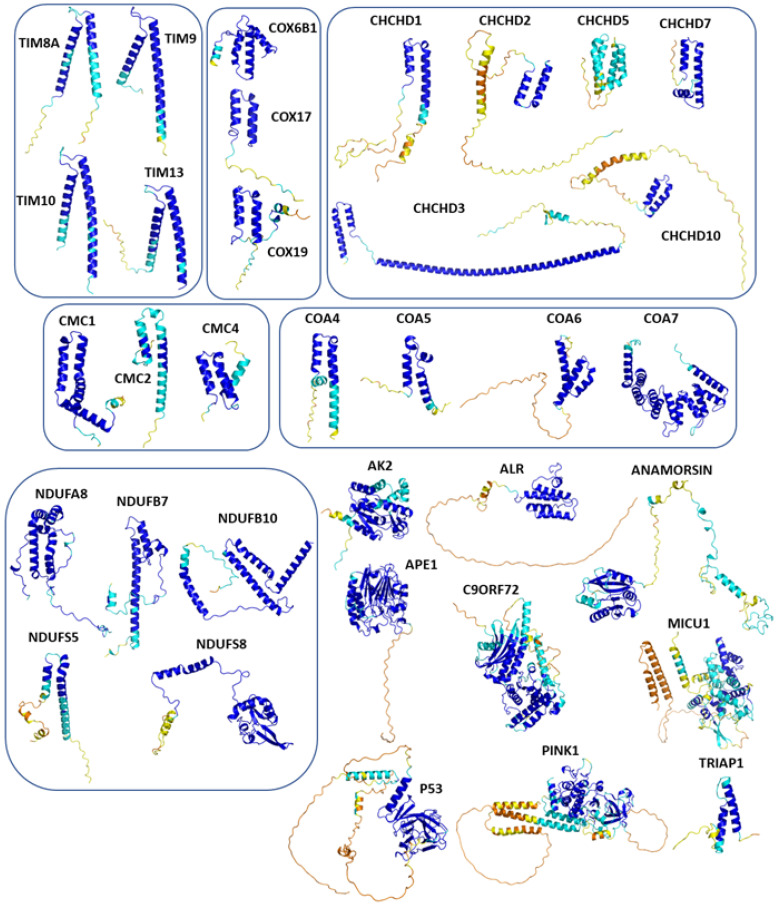
AF3 predicted structures of all hitherto identified hCHCH4 substrates. The cartoons have been colored according to the AF3 per-residue confidence metric (pLDDT), which adopts the following scale: blue (very high confidence) for pLDDT > 90, cyan (confident) for 70 < pLDDT ≤ 90, yellow (low confidence) for 50 < pLDDT ≤ 70, and orange (very low confidence) for pLDDT < 50. In line with the AF3 self-assessment, regions with low pLDDT values (yellow and orange) correspond to flexible protein fragments that likely present dynamic behaviors for which the predicted structure is virtually meaningless.

The survey of the literature data indicates that hCHCHD4/yMia40 operates on a multitude of substrates that have often quite different three-dimensional structures (Figure 1), and whose interactions with the enzyme have been differently characterized.

Considering the information retrieved from the literature for each substrate, they were classified into the following three distinct groups (see Table 2), based on the availability of experimental data concerning the docked cysteine. The first one includes well-established substrates whose interaction with hCHCHD4/yMia40 has been the subject of structural investigations (Group I). The second group includes the substrates that have been experimentally validated, but without any structural information available. This ensemble has been split into two sub-groups depending on whether the docked cysteine, i.e., the one involved in the covalent binding with hCHCHD4/yMia40, was identified (Group IIa) or not (Group IIb). Within each group, the substrates were grouped in families when more than one member was a validated partner of hCHCHD4. In collecting literature data, for each substrate, we have reported, if known, the reactive Cys residue.

**Table 2:** List of redox-regulated substrates of hCHCHD4, along with their identification code (UniprotKB), yeast homolog, and some global parameters (length, CHCHD4 recognition motif, docked Cys residues). The proteins are divided into two groups. Group I includes substrates for which structural characterizations have been reported in the literature. Group II consists of those substrates that have been biochemically characterized. Group II is further divided into subgroups IIa and IIb. Subgroup IIa includes substrates for which the cysteine involved in the covalent binding with hCHCHD4 has been identified. Subgroup IIb includes those for which no such data have been reported.

Name	UniprotKB	Yeast Homolog	Length (aa)	The Recognized Cysteine Motif (CX_n_C)_2_	Docked Cysteine Residue on the Substrates	Reference
**GROUP I**						
ALR	P55789	Erv2	205	CX_2_C-X_15_-CX_16_C	C71 or C74	[50]
COX17	Q14061	Cox17	67	CX_9_C-X_8_-CX_9_C	C45	[49]
TIMM9	Q9Y5J7	Tim9	89	CX_3_C-X_15_-CX_3_C	* C35 ** C28	[49]
TIMM10	P62072	Tim10	90	CX_3_C-X_16_-CX_3_C	* C35 ** C29	[49]
**GROUP IIa**						
AK2	P54819	-	239	-	C42	[67,118]
APE1	P27695	-	318	-	C93	[113,126]
CHCHD2 (MNRR1)	Q9Y6H1	Mix17	151	CX_9_C-X_9_-CX_9_C	C144	[67,129]
CHCHD3 (MIC19)	Q9NX63	Mic19	227	CX_9_C-X_10_-CX_9_C	C193	[67,112,130,131]
CMC1	Q7Z7K0	Cmc1	106	CX_9_C-X_11_-CX_9_C	* C42 ** C31	[112,121]
COX19	Q49B96	Cox19	90	CX_9_C-X_10_-CX_9_C	C51	[39]
MICU1	Q9BPX6	-	476	-	* C463 ** C463	[127]
NDUFB10	O96000	-	172	CX_6_C-X_29_CX_11_C	C107	[117]
**GROUP IIb**						
Anamorsin	Q6FI81	Dre2	312	-	-	[101]
C9orf72	Q96LT7	-	481	CX_2_C-X_29_-CX_3_C	-	[132,133]
CHCHD1	Q96BP2	Mrp10	118	CX_9_C-X_10_-CX_9_C	-	[125]
CHCHD5	Q9BSY4	Mic14	110	CX_9_C-X_11_-CX_9_C-X_13_-CX_9_C-X_10_-CX_9_C	-	[110,112]
CHCHD7 (COX23)	Q9BUK0	Cox23	85	CX_9_C-X_10_-CX_9_C	-	[110]
CHCHD10	Q8WYQ3	Mic17	142	CX_9_C-X_9_-CX_9_C	-	[134]
CMC2	Q9NRP2	Cmc2	79	CX_9_C-X_12_-CX_9_C	-	[85]
CMC4	P56277	Cmc4	68	CX_9_C-X_9_-CX_9_C-CX_10_C	-	[112]
COA4 (CMC3)	Q9NYJ1	Coa4	87	CX_9_C-X_9_-CX_9_C	-	[67,72,112]
COA5 (PET191, C2orf64)	Q86WW8	Pet191	74	CX_9_C-X_22_-CX_9_C CX_9_C-X_5_-CX_9_C-X_5_-CX_9_C	-	[112]
COA6 (C1orf31)	Q5JTJ3	Coa6	125	CX_9_C-X_10_-CX1_0_C	-	[112]
COA7	Q96BR5	-	231	-	-	[135]
Cox6B1	P14854	Cox12	86	CX_9_C-X_13_-CX_10_C	-	[112]
NDUFA8	P51970	-	172	CX_9_C-X_9_-CX_9_C-X_11_-CX_9_C-X_10_-CX_9_C	-	[72]
NDUFB7	P17568	-	137	CX_9_C-X_10_-CX_9_C	-	[67,136]
NDUFS5	O43920	-	106	CX9C-X12-CX9C	-	[67,112]
NDUFS8	O00217	-	210	CX_9_C-X_28_-CX_9_C	-	[112]
p53	P04637	-	393	CX_5_C-X_134_-CX_2_C	-	[137]
PINK1	Q9BXM7	-	581	-	-	[138,139]
TIMM8A	O60220	Tim8	97	CX_3_C-X_14_-CX_3_C	-	[19]
TIMM13	Q9Y5L4	Tim13	95	CX_3_C-X_15_-CX_3_C	-	[53,112]
TRIAP1 (MDM35)	O43715	Mdm35	76	CX_9_C-X_18_-CX_9_C	-	[140]

* Refers to the docked cysteine identified in the yeast substrates. ** Refers to the putative docked cysteine in the human homolog of the substrates.

### 4.1. The TIMM Protein Family

Mitochondrial proteins featuring the well-conserved (CX_3_C)_2_ motifs include the Tim proteins, key chaperones involved in the general mitochondrial protein import pathway, that have been extensively studied as redox substrates of yMia40 [56,90,92,93,98,141,142]. Studies on Tim9 and Tim10 have successfully identified both the specific sequences responsible for their interaction with yMia40 and the crucial Cys residue involved in disulfide bonding, as the one located upstream of the ITS. This has led to establishing a consensus sequence for this protein family directly involved in the binding of yMia40 [47,48,92,93]. In contrast, the characterization of human homologs has been limited. Preliminary evidence regarding the role of hCHCHD4 in the biogenesis of human Tim proteins, collectively referred to as TIMMs, emerged in 2005 [53]. It was demonstrated that the depletion of hCHCHD4 specifically reduced the steady-state levels of small, cysteine-rich intermembrane space proteins, including TIMM8A (also known as DDP1) and TIMM10A, in human cells [53]. Similarly, the downregulation of AIF, which disrupts the mitochondrial import mediated by hCHCHD4, adversely affects the biogenesis of TIMM8A [19]. More recently [112], a quantitative proteomic study showed that the total levels of proteins such as TIMM9, TIMM10, and TIMM13 were significantly elevated in cells expressing the wild-type hCHCHD4. Conversely, cells expressing the redox-inactive mutant CHCHD4C53S exhibited reduced levels of these proteins [112]. Additionally, Banci et al. (2010) characterized the interaction between hCHCHD4 and yeast Tim10 (yTim10), and hCHCHD4 and a peptide from yeast Tim9 (yTim9) that mimics the ITS region using NMR techniques [49] (see below).

### 4.2. The COX Protein Family

The members of the human cytochrome c oxidase (COX) family, which are substrates for the hCHCHD4 oxidoreductase, are essential factors for the biogenesis and assembly of respiratory chain complex IV (CIV), and their deficiency is a common cause of mitochondrial diseases [143,144,145,146,147,148]. Numerous studies have demonstrated the direct interactions between yMia40 and the yeast proteins Cox17 and Cox19 [22,91,143,149]. Additionally, the interaction between hCHCHD4 and COX17/19 has been substantiated in human cells [19,39,48,72,112]. NMR analyses further indicate that human COX17 forms an intermolecular disulfide bond with hCHCHD4 [39,49,150]. Importantly, specific cysteine residues downstream of the ITS have been implicated in this interaction (Table 2). In these proteins, a well-conserved recognition motif (CX_9_C)_2_ has been detected. More recently, for the Cox6B1 (Cytochrome c oxidase subunit 6B1), quantitative proteomic analyses, corroborated by immunoblotting on total lysates, revealed a reduction in protein levels in cells expressing singly mutated hCHCHD4 C53S and C55S, doubly mutated C53S-C55S hCHCHD4, as well as in CHCHD4 CRISPR-Cas9 knockout clones [112]. Notably, this protein does not contain a twin CX_9_C motif (see Table 2).

### 4.3. The CMC Protein Family

Other proteins containing the (CX_9_C)_2_ motif include the COX assembly mitochondrial protein homologs CMC1 and CMC2, which are related to the biogenesis of complex IV (CIV), and CMC4, a protein with an unknown function (Table 1) [108]. Various studies in yeast have documented the dependency of Cmc1, Cmc2, Cmc3, and Cmc4 on yMia40 for their mitochondrial translocation and/or biogenesis [108,121,151,152]. Specifically, based on mutational studies, it has been hypothesized that Cys42 in Cmc1 is involved in its interaction with yMia40 [121]. In mammalian cells, all these proteins have been identified as substrates of hCHCHD4 through a proteomic approach, and their overall levels in cells have been correlated with the expression levels of the wild-type and/or of redox mutants of hCHCHD4 [112]. Furthermore, experiments in mammalian cells have shown that the overexpression of CMC3 delays the oxidation of COX19, suggesting a potential competitive interaction for oxidation-dependent import [119]. It should be noted that CMC4, in addition to the canonical CX_9_C motif, also has the CX_10_C motif, and the two cysteine residues of this motif are engaged in a disulfide bridge (see Table 4 in Section 6).

### 4.4. The CoA Protein Family

Cytochrome c oxidase assembly factors COA4, COA5, COA6, and COA7 [72,135,153,154,155], which are associated with the assembly of CIV [72], are identified as substrates of hCHCHD4 (Table 2) [112]. As reported for the CMC proteins, the overall levels of COA proteins in cells have been correlated with the expression levels of the wild-type and/or redox mutants of hCHCHD4 [112].

Moreover, affinity purification experiments, combined with immunoblotting and mass spectrometry analysis, revealed a direct interaction between the cytochrome c oxidase assembly factor 7 (COA7) and hCHCHD4. Unlike typical hCHCHD4 substrates, COA7, present in human but not in yeast, possesses 13 cysteine residues that that are not organized according to the common hCHCHD4 targeting signals, such as the ITS, nor do they align with traditional motifs like CX_3_C or CX_9_C [90,92], highlighting the diversified role of hCHCHD4 in the redox pathways of various proteins, compared to yMIA40.

### 4.5. The CHCHD Protein Family

The mitochondrial CHCHD proteins belong to the class of (CX_9_C)_2_-containing motif proteins. They play important roles in the pathophysiology of mitochondria and other essential cellular processes [156,157,158,159,160,161,162,163,164,165]. In yeast, they are generally imported into the IMS of the mitochondria via a disulfide relay system that involves the proteins yMia40 and Erv1 [125,166]. However, the precise molecular mechanisms governing the cell-specific regulation of their import and accumulation within mitochondria, particularly through the hCHCHD4-dependent import machinery, have yet to be fully elucidated. Among the CHCHD proteins identified as substrates of hCHCHD4 (Table 2), the interaction of CHCHD2, CHCHD3, and CHCHD10 with hCHCHD4 has been characterized in more detail [67,112,129,130,167]. CHCHD2, also known as MNRR1, is predominantly found in the mitochondria, although a portion is present in the nucleus. Within mitochondria, CHCHD2 plays a crucial role in the functioning of cellular respiration, regulating the production of reactive oxygen species (ROS), and managing oxidative stress. Cells with diminished levels of CHCHD2 exhibit clear signs of mitochondrial dysfunction [129,168,169,170,171,172,173,174]. It was found that mammalian endogenous hCHCHD4 co-immunoprecipitates with CHCHD2, and mutational analysis of cysteine residues in CHCHD2, combined with co-immunoprecipitation experiments, has identified Cys144 as the key docking residue [129]. A direct interaction has also been established between CHCHD3 and hCHCHD4 [130]. CHCHD3, a myristoylated mitochondrial protein, forms a transient disulfide-bonded intermediate primarily between the second cysteine in helix 1 (Cys193) and the active site cysteine of hCHCHD4 (Cys55) [130]. Truncation experiments indicate that the mitochondrial import of CHCHD10 is primarily facilitated by its CHCH domain, rather than the mitochondrial targeting signal identified at its N-terminus [134]. Notably, the knockdown of hCHCHD4 blocks the mitochondrial import of CHCHD10, whereas its overexpression effectively restores the mitochondrial import of the CHCHD10 Q108P variant, a mutation within the CHCH domain that nearly completely blocks the mitochondrial import [134]. The mutation of Cys122, identified in amyotrophic lateral sclerosis (ALS) cases [134], disrupts the mitochondrial import of CHCHD10, emulating the effects observed with the Q108P mutation. Not only does this finding reinforce the importance of disulfide bond formation within the CHCH domain for successful mitochondrial import, but it also implies that Cys122 may be involved in establishing an inter-disulfide bond with hCHCHD4 [134].

### 4.6. The NDUF_NADH Dehydrogenase Protein Family

Several subunits of complex I that contain the (CX_9_C)_2_ motif, including NDUFB7, NDUFS5, NDUFA8, and NDUFS8, have been identified as potential substrates for hCHCHD4 (Table 2) [67,112,136]. Notably, these proteins are present in humans but not in yeast. NDUF proteins have been identified as potential substrates in proteomic studies since their levels correlate with the presence of either wild-type or mutants of hCHCHD4 as well as the presence of AIF [67,112]. Among them, NDUFA8 presents a (CX_9_C)_2_ motif, and its mitochondrial import kinetics are modulated by hCHCHD4 and ALR [72]. Interestingly, NDUFB10, a newly identified hCHCHD4 substrate, is an accessory subunit of complex I, characterized by an unusual cysteine motif (CX_6_C/CX_11_C). A mutation in the conserved Cys107 of NDUFB10 has been linked to a severe CI-related mitochondrial disease and has been shown to inhibit both the hCHCHD4-dependent oxidation of the protein and its accumulation within mitochondria [117]. Beyond these potential substrates of hCHCHD4, other protein subunits of the respiratory chain complex I seem to be regulated by the hCHCHD4-dependent import pathway. For example, the depletion of AIF negatively impacts the expression of nucleus-encoded subunits of complex I, including NDUFA9, NDUFS7, NDUFB6, NDUFB8, and NDUFA13, in mammalian cells [19].

### 4.7. The Human Adenylate Kinase 2

The human Adenylate kinase 2 (AK2) plays a vital role in reversible phosphoryl transfer among adenine nucleotides, specifically by catalyzing the reaction 2ADP ↔ ATP + AMP in the IMS [175]. The disulfide bond formation between Cys42 and Cys92 is crucial for mitochondrial AK2 accumulation. Unlike classical hCHCHD4 substrates, AK2 is an unconventional redox-regulated substrate for its odd number of conserved cysteines (Cys40, Cys42, Cys92) and their atypical spacing (Table 2). Only Cys40 and Cys92 are embedded in helices and serve as classical hCHCHD4 interaction targets. The lack of these two cysteine residues leads to the mislocalization of the protein, thus indicating that they play a role in mitochondrial accumulation. Immunoprecipitation experiments show that the mutation of Cys40 disrupts the AK2–hCHCHD4 interactions and delays the oxidation kinetics [67,118]. This finding confirms that an initial mixed disulfide bond is formed by Cys40 and hCHCHD4, which may then isomerize to the final disulfide bond between Cys42 and Cys92. In vitro experiments indicated that a labile disulfide bond could be formed between Cys40 and Cys42, whose limited stability may favor the rearrangement toward a stable Cys42–Cys92 bond [118].

### 4.8. The Protein p53

Traditionally recognized as a nuclear protein, p53 is vital for maintaining genomic integrity and regulating gene expression. However, several investigations have also highlighted its role in the repair and maintenance of mitochondrial DNA [176,177]. Interestingly, the function of p53 is sensitive to redox conditions, with the oxidation of its cysteine residues affecting its subcellular localization [178]. Specifically, p53, which does not possess a canonical (CX_n_C)_2_ motif, contains two pairs of cysteines (Cys135–Cys141 and Cys275–Cys277) that can form intramolecular disulfide bonds, thus suggesting that this protein may represent a substrate for hCHCHD4 under conditions of elevated oxidative stress (Table 2). In this scenario, in 2013, it was shown that p53 and hCHCHD4 colocalize and directly interact within the mitochondria, as evidenced by confocal imaging and pull-down assays [14,137]. Furthermore, this interaction is closely linked to the redox activity of hCHCHD4. This interplay modulates the subcellular partitioning of p53 between the nucleus and mitochondria and affects its nuclear activity. The interaction between p53 and hCHCHD4 has also been detected in vivo [14,137,179].

### 4.9. The Protein TRIAP1

TRIAP1, also known as TP53-Regulated Inhibitor of Apoptosis or p53CSV, is a small (about 8 kDa) mitochondria-shaping protein with a canonical (CX_9_C)_2_ motif that regulates the phospholipid trafficking between outer and inner membranes, and was revealed to be involved in p53-mediated stress response pathways [180,181,182,183,184,185,186,187,188,189]. Its three-dimensional structure is characterized by an antiparallel helix–loop–helix motif linked by two intramolecular disulfide bonds, designated as the inner (Cys18-Cys37) and the outer disulfide (Cys8-Cys47), followed by a disordered C-terminal region [190]. Studies on its yeast homolog, Mdm35, indicate that TRIAP1 requires a disulfide relay for the mitochondrial import [11,108,166]. In mammalian cells, two independent reports revealed a correlation between the levels of hCHCHD4 and TRIAP1, indicating a specific interaction between these two proteins [112,191]. Moreover, TRIAP1 was found to be one of the proteins most affected by hCHCHD4 haploinsufficiency since it shows a marked decrease in heart mitochondria in hCHCHD4 heterozygous knockout (hCHCHD4+/−) mice [191]. In vivo studies have demonstrated that TRIAP1 levels decreased over time upon a reduction in hCHCHD4 in the skeletal muscle of wild-type mice after acute exercise. This decline in TRIAP1 was prevented in transgenic mice that maintained elevated hCHCHD4 levels [191]. Very recently, it has been reported in vitro that hCHCHD4 facilitates the oxidation of the inner disulfide bond between Cys18 and Cys37 of TRIAP1, and subsequently catalyzes the formation of the outer disulfide bond between Cys8 and Cys47 to induce the native structure of the protein [140].

### 4.10. The Protein Anamorsin

Human Anamorsin, the homolog of the yeast Dre2, is involved in the biogenesis of cytosolic iron–sulfur (Fe/S) proteins and was the first Fe/S protein identified to be imported into the IMS of mitochondria [101]. Structural analyses reveal that Anamorsin consists of two independent domains connected by an unstructured region, with the C-terminal domain binding a [2Fe-2S] cluster through a unique cysteine-rich motif. Anamorsin does not contain a canonical (CX_n_C)_2_ motif (see Table 2). Both Anamorsin and Dre2 have been shown to physically interact with yMia40 and hCHCHD4 in vitro; for Dre2, its interaction with yMia40 has also been validated in mitochondria of *Saccharomyces cerevisiae*. yMia40/hCHCHD4 specifically oxidizes the twin CX_2_C motif of Dre2/Anamorsin without affecting the Fe/S cluster-binding capabilities [101].

### 4.11. The Protein C9orf72

C9orf72 is a protein associated with the inner mitochondrial membrane, where it plays a vital role in regulating cellular energy homeostasis due to its impact on the oxidative phosphorylation process [132,192].

Proteomic studies and co-immunoprecipitation assays have identified AIF as a binding partner of C9orf72 [132]. This interaction likely drives its mitochondrial localization. Interestingly, the analysis of C9orf72 from the mitochondrial fraction reveals a partially oxidized state, whereas the protein is fully reduced in the cytosol [132]. The involvement of hCHCHD4 in the oxidation process of C9orf72 is suggested by the silencing of either AIF or hCHCHD4, which leads to a significant reduction in C9orf72 levels within the mitochondrial fraction. Furthermore, triple mutations of C9orf72 targeting either side of the cysteine cluster completely abolished its mitochondrial localization [132].

### 4.12. The Protein APE1

APE1 (apurinic/apyrimidinic endonuclease 1) is a multifunctional protein that plays a crucial role in repairing lesions in nuclear and mitochondrial DNA caused by oxidative and alkylating agents [193,194]. APE1 does not contain a canonical (CX_n_C)_2_ motif, although recent findings have shown that APE1 interacts in mammalian cells with hCHCHD4 [113], thus indicating a potential redox-assisted mechanism for the transfer of APE1 in the mitochondria. This consideration is corroborated by the observation that APE1 and hCHCHD4 engage through the formation of disulfide bonds, as demonstrated by immunoprecipitation experiments and pull-down assays in reducing and non-reducing conditions [113]. Moreover, the levels of hCHCHD4 expression significantly influence the translocation of APE1 to mitochondria. The mitochondrial targeting signal of APE1 has been identified in the region encompassing residues 289–318, with Lys299 and Arg301 identified as critical sites [113,126]. Mutations at these positions completely abolish the APE1 mitochondrial translocation during oxidative stress. Once in the mitochondria, the formation of the bridge between Cys93 of APE1 and Cys55 of hCHCHD4 is observed by GST pull-down assays. Interestingly, when Cys65, the partner of Cys93 in the functional disulfide of APE1, is mutated to Ser, an enhanced interaction between the two proteins is detected [126]. This finding suggests that the oxidation reaction is trapped in the intermediate state characterized by the formation of the heterodimer covalent adduct [126].

### 4.13. The Protein PINK1

Recent studies have identified PINK1 (PTEN-induced putative kinase 1) as a substrate of hCHCHD4 [33,138]. This protein is a crucial factor for the PINK1-mediated mitophagy that requires its accumulation on the outer mitochondrial membrane [139]. Structural analyses have evidenced a putative outer mitochondrial membrane localization signal in the PINK1 sequence, located between amino acids 74 and 93 [195]. The deletion of this region hinders the retention of PINK1 in damaged mitochondria, impeding mitophagy. Notably, the Cys92 may facilitate disulfide bond formation upon mitochondrial damage, potentially preventing PINK1’s mitochondrial import and full processing, thus promoting its accumulation on the outer mitochondrial membrane [33]. Additionally, an amphipathic helix (amino acids 166–172) within PINK1, which may interact with hCHCHD4, is vital for its binding and accumulation [196]. The deletion of this helix significantly decreases the interaction of PINK1 with hCHCHD4 [33,138]. Notably, the mutations A168P and V170G, related to Parkinson’s disease (PD), also impair the interaction of PINK1 with hCHCHD4 [138].

### 4.14. The Protein MICU1

MICU1 is a substrate of hCHCHD4 localized in the IMS that is essential for mitochondrial Ca^2+^ uptake [127,197]. MICU1 presents several distinctive characteristics compared to previously identified hCHCHD4 substrates. Firstly, it possesses a mitochondrial targeting sequence (MTS), enabling its import into mitochondria without the need for hCHCHD4. Moreover, MICU1 contains seven cysteine residues, with only Cys463 being conserved. In vitro experiments have shown that MICU1 cysteine mutants successfully formed disulfide-linked dimers with hCHCHD4, except for the variant MICU1C463A. In particular, the heterodimerization of MICU1 with hCHCHD4 results in the complex formation of MICU1 and MICU2, as the redox-active Cys55 of hCHCHD4 transiently interacts with Cys463 in MICU1, which then forms a mixed disulfide bond with a cysteine residue in MICU2 [127].

### 4.15. The Thiol Oxidase ALR

The protein Augmenter of Liver Regeneration (ALR), the human homolog of the *S. cerevisiae* enzyme Erv1, is a flavin adenine dinucleotide FAD-dependent sulfhydryl oxidase, and operates as a critical regulatory element in the oxidative folding of proteins within the IMS [17,52,122,123]. It plays a pivotal role in the reoxidation of the CPC motif in hCHCHD4, which is essential for its recycling. Studies conducted in yeast allowed the inclusion of Erv1 (the yeast homolog of ALR), which carries a CX_2_C-X_15_-CX_16_C motif (Table 1), in the list of atypical yMia40 substrates [11,17,198]. Here, we discuss ALR since the mechanism of formation of the hCHCHD4-ALR complex involves Cys55 of hCHCHD4 and, therefore, likely resembles that detected for substrates.

In yeast, the intrinsically disordered N-terminal segment of Erv1, which features a CX_2_C motif, facilitates the reoxidation of the CPC motif [199]. In human cells, Fischer et al. [72] demonstrated, through a protein oxidation assay, that the concomitant import and oxidative folding of hCHCHD4 substrates relies on hCHCHD4 oxidase activity as the primary catalytic component and on ALR, the hCHCHD4 recycling partner. This process depends on the interaction of ALR with the hydrophobic groove of hCHCHD4 for its effective reoxidation [50]. The disruption of this interaction could impede reoxidation and result in a less oxidized CPC motif. ALR is specifically targeted for the nucleophilic attack by Cys55 during hCHCHD4 reoxidation. Data from the literature suggest that the reduced efficiency of the hCHCHD4F68E variant, in which the ARL/substrate recognition motif is perturbed, could be ascribed to both the impaired reoxidation of hCHCHD4 by ALR and the destabilization of the disulfide-linked hCHCHD4–substrate complex [50].

## 5. The Structural Basis of the hCHCHD4 Redox-Regulated Substrate Recognition

Despite the key functional role of hCHCHD4 in several contexts and the diversity of its substrates, atomic-level information on the recognition process is still rather limited. A survey of the three-dimensional structures of hCHCHD4 and its homolog reported in the PDB (March 2025 release) indicates that only six models have hitherto been deposited.

Chronologically, the first structure deposited for this protein corresponds to the NMR structure of the folded domain (residues 41–105) of hCHCHD4 (PDB code 2K3J) [47,53]. In the structure of this domain, two disulfide bridges (Cys64-Cys97 and Cys74-Cys87) are observed, while the reactive Cys55 of the CPC is either in a reduced state or oxidized, which is achieved by forming a bridge with Cys53, depending on the presence of reducing agents. As expected, the formation of the Cys53-Cys55 disulfide bond limits the flexibility of the CPC fragment. Globally, the hCHCHD4 structure resembles a fruit-dish-like shape with approximate dimensions of 20 Å × 27 Å × 36 Å. In terms of secondary structure elements, the folded core of hCHCHD4 contains two α-helices (residues 64–79 and 87–101) connected by a relatively long loop. This antiparallel α-hairpin motif is followed by an unstructured C-terminal region and is stabilized by the two disulfide bridges mentioned above. Similar structural elements are exhibited by yMia40, whose crystallographic structure has been reported as a fusion protein with the Maltose Binding Protein (PDB codes 2ZXT and 3A3C) [80]. The inspection of the PDB also revealed the presence in the database of three complexes involving hCHCHD4 (Table 3).

**Table 3:** Experimental structures of available mammalian CHCHD4 and yMia40 in the PDB.

Protein (Length, aa)	Organism (UniProtKB)	PDB Entry	Method (Resolution, Å)	Protein Region (Residues)	Notes	Reference
hCHCHD4 (142)	*H. sapiens* (Q8N4Q1)	2K3J	NMR	41–105	hCHCHD4 residues 1–40 and 106–142 are missing *. C53S mutation.	[47]
		2L0Y	NMR	46–105	Complex of hCHCHD4 and human cytochrome c oxidase copper chaperone COX17 (UniProtKB Q14061, residues 1–63). hCHCHD4 residues 1–45 and 106–142 and COX17 residues 1–42 are missing *. C55S mutation in COX17.	[49]
		8VGY	X-ray diffraction (2.30)	1–30	Fusion protein of human apoptosis-inducing factor 1 AIFM1 (UniProtKB O95831, residues 104–613), Linker (SGSGPGSGS), hCHCHD4 (residues 1–45). AIFM1 residues 104–126 and 512–557 and hCHCHD4 residues 31–45 are missing *. W196A mutation in AIFM1.	[66]
yMia40 (403)	*S. cerevisiae* (P36046)	2ZXT	X-ray diffraction (3.00)	284–353	Fusion protein of maltose-binding periplasmic protein MMBP (UniProtKB P0AEX9, residues 27–392), Linker (NSSSVPGRGSIEGRPEF), yMia40 (residues 284–365). yMia40 residues 354–365 are missing *.	[80]
		3A3C	X-ray diffraction (2.50)	284–353	Fusion protein of MMBP (UniProtKB P0AEX9, residues 29–392), Linker (NSSSVPGRGSIEGRPEF), yMia40 (residues 284–365). yMia40 residues 354–365 are missing *. C296S, C298S mutations in yMia40.	
mCHCHD4 (139)	*M. musculus* (Q8VEA4)	8QNS	X-ray diffraction (3.21)	4–14	Complex of *M. musculus* apoptosis-inducing factor 1 Aifm1 (UniProtKB Q9Z0 × 1, residues 101–612) and mCHCHD4 (residues 1–27). Aifm1 residues 101–124 and 512–556 and mCHCHD4 residues 1–3 and 15–27 are missing *.	[200]

* Missing residues mentioned in the Notes, although present in the expressed proteins, are not reported in the coordinate files due to their extreme flexibility.

Two X-ray structures correspond to the recently determined complexes formed by AIF with the N-terminal peptide of mCHCHD4 [200] and hCHCHD4 [66], which are not related to the catalytic role of the protein, and therefore, are not relevant for this study. The other PDB entry reports the structure of the complex that hCHCHD4 forms with COX17 that presents the canonical (CX_9_C)_2_ motif [49]. Although not reported in the PDB, structural information on the interactions between hCHCHD4 and redox substrates is present in the literature. In particular, structural data are reported for the complexes formed by the protein with yeast Tim10 (yTim10) and a yeast Tim9 peptide (yTim9) mimicking the intermembrane space targeting signal (ITS) region [49], both presenting the (CX_3_C)_2_ motif.

**Figure 2:**
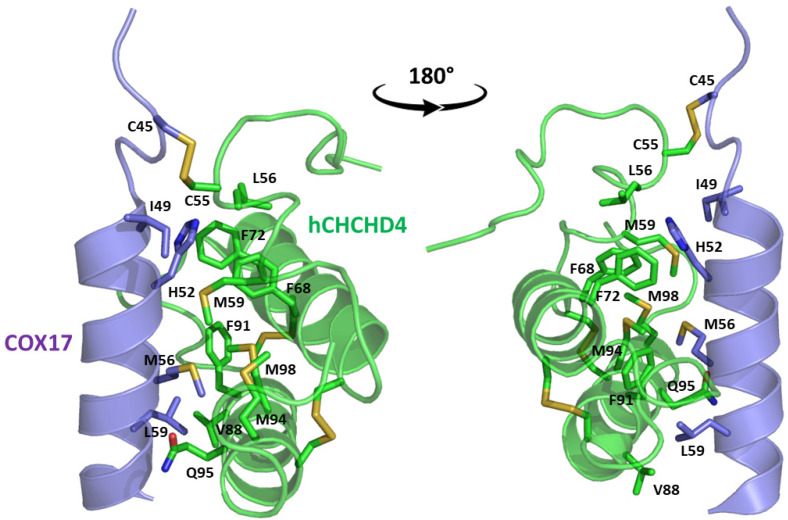
Two views of the complex formed by hCHCHD4 (green) with a peptide of the COX17 protein (violet). The figure has been generated using the coordinates reported in the PDB entry 2L0Y. Residues involved in the protein–protein interface are shown with a stick representation.

In these studies, as a general strategy, to avoid the formation of uncontrolled and non-physiological disulfides, some cysteine residues of the enzyme (Cys53) and the substrates were mutated to serine residues. Collectively, these data indicate that the oxidative reaction proceeds with distinct steps. The initial event is the protein–protein recognition that is essential to select and correctly position the cysteine residues that will be involved in the formation of the intermolecular disulfide bond. The mutual approach of the two proteins favors the formation of an amphipathic helix in the ITS region of the substrate. The apolar face of the ITS helix interacts with the hCHCHD4 hydrophobic residues, thus reinforcing the correct pairing of the reactive Cys residues (see Figure 2 for a representative example). After the formation of the interchain disulfide bond, the putative nucleophilic attack of the second cysteine of the substrate to the disulfide bond leads to the formation of the intramolecular disulfide bond, which then allows the release of the substrate. This final event also favors the folding of the second helix of the substrate, which assumes its final oxidized structure. This cycle restarts with the reoxidation of hCHCHD4 by its recycling partner ALR. Interestingly, it has been proposed that the recognition mode of hCHCHD4 by ALR resembles the interactions observed in the docking of the substrates with hCHCHD4 [50]. Indeed, also in this case, the recognition process is driven by the hydrophobicity present in specific regions of the two proteins.

## 6. Extensive Analysis of the Structures of hCHCHD4 Substrates: The Contribution of AlphaFold

In contrast to the paucity of structural information collected for complexes formed by hCHCHD4, extensive structural data are available for the three-dimensional organization of the substrates. Indeed, as illustrated below, the integration of the experimental data reported in the PDB for these proteins with those that could be retrieved from the EBI-AlphaFold dataset or generated by using the AlphaFold3 server provides a complete picture of their three-dimensional structures (Figure 1). These data show the ability of the hCHCHD4 protein to operate on quite different substrates. Moreover, these predictions also provide information on the structural context of the docked Cys, not only for substrates of Group IIa but occasionally for members of Group IIb (see below for details).

It is important to note that the use of AlphaFold provides atomic-level structural information for the following proteins for which no experimental structure is present in the PDB: COX19, CHCHD2, CHCHD3, CHCHD10, CMC1, CMC2, COA4, and COA5. Notably, for all these proteins, no information is available for homologs either, at least using the Blast search scores (https://blast.ncbi.nlm.nih.gov/, accessed on 1 March 2025). For PINK1, AF3 provides the structure of the human protein, whereas in the PDB, some structures of non-mammalian homologs are reported. In Table 4, the three-dimensional structures of these proteins are reported. When a PDB model was available, it was superimposed on the AF3 one. A quick inspection of these fittings and the related root mean square deviations (RMSD) values, computed on the C^α^ atoms, provides clear evidence of the ability of AF3 to predict these structures.

In the next paragraphs, brief descriptions of the folds of all thirty-four substrates hitherto identified are provided, along with the local environment of the Cys residue recognized by hCHCHD4. These data are illustrated *per* family and for individual substrates following the scheme reported in Section 4 (Figure 1).

**Table 4:** Redox substrates of hCHCHD4. The table shows the AlphaFold-predicted three-dimensional models of the redox-substrates of hCHCHD4, and, if available, the superimposition of a selected experimental structure (in magenta) with the RMSD values (computed on the C^α^ atoms). In addition, the disulfide bridges, the docked Cys residues (shown as a yellow sphere for groups I and IIa), the amino acid sequences (with Cys residues in red), the PDB codes of the experimental structures, and the PAE matrices (see the text for the definition) of predicted models are reported.

Substrate (UniprotKB)	Disulfide Bridges, Docked Cys, Sequence	PDB Structure *	Superimposition (Exp vs. AF) or AF3 Model	PAE of the AF3 Model
**Group I**				
ALR (P55789)	C142-C145, C171-C188 Docked Cys: C71 or C74 MAAPGERGRFHGGNLFFLPGGARSEMMDDLATDARGRGAGRRDAAASASTPAQAPTSDSPVAEDASRRRPCRACVDFKTWMRTQQKRDTKFREDCPPDREELGRHSWAVLHTLAAYYPDLPTPEQQQDMAQFIHLFSKFYPCEECAEDLRKRLCRNHPDTRTRACFTQWLCHLHNEVNRKLGKPDFDCSKVDERWRDGWKDGSCD	Code: 3U5S Technique: X-ray crystallography (Resol. 1.50 Å) Chain: A Residues: 82–203	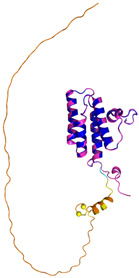 RMSD: 0.26 Å (114 C^α^ aligned)	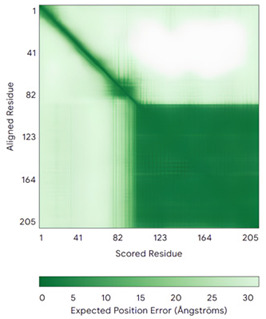
COX17 (Q14061)	C26-C55, C36-C45 Docked Cys: C45 MPGLVDSNPAPPESQEKKPLKPCCACPETKKARDACIIEKGEEH**C**GHLIEAHKECMRALGFKI	Code: 2RNB Technique: NMR Chain: A Residues: 1–63	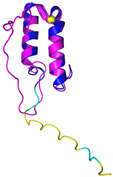 RMSD: 0.64 Å (38 C^α^ aligned)	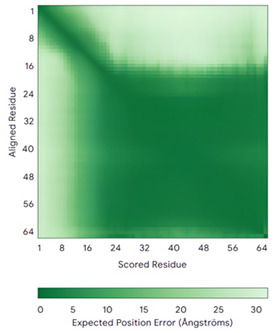
TIMM9 (Q9Y5J7)	C28-C52, C32-C48 Docked Cys: C28 ESDQIKQFKEFLGTYNKLTETCFLDCVKDFTTREVKPEETTCSEHCLQKYLKMTQRISMRFQEYHIQQNEALAAKAGLLGQPR	Code: 2BSK Technique: X-ray crystallography (Resol. 3.30 Å) Chain: A Residues: 1–89	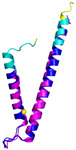 RMSD: 0.78 Å (67 C^α^ aligned)	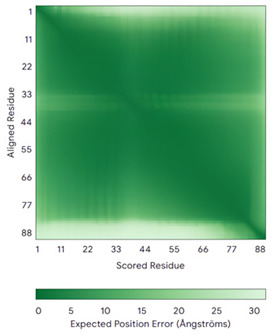
TIMM10 (P62072)	C29-C54, C33-C50 Docked Cys: C29 MDPLRAQQLAAELEVEMMADMYNRMTSACHRKCVPPHYKEAELSKGESVCLDRCVSKYLDIHERMGKKLTELSMQDEELMKRVQQSSGPA	Code: 2BSK Technique: X-ray crystallography (Resol. 3.30 Å) Chain: B Residues: 1–90	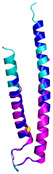 RMSD: 0.90 Å (60 C^α^ aligned)	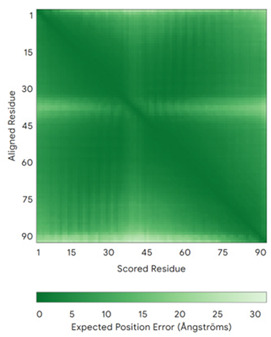
**Group IIa**				
AK2 (P54819)	C42-C92 Docked Cys: C42 MAPSVPAAEPEYPKGIRAVLLGPPGAGKGTQAPRLAENFCVCHLATGDMLRAMVASGSELGKKLKATMDAGKLVSDEMVVELIEKNLETPLCKNGFLLDGFPRTVRQAEMLDDLMEKRKEKLDSVIEFSIPDSLLIRRITGRLIHPKSGRSYHEEFNPPKEPMKDDITGEPLIRRSDDNEKALKIRLQAYHTQTTPLIEYYRKRGIHSAIDASQTPDVVFASILAAFSKATCKDLVMFI	Code: 2C9Y Technique: X-ray crystallography (Resol. 2.10 Å) Chain: A Residues: 1–239	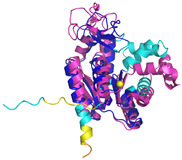 RMSD: 2.9 Å (180 C^α^ aligned)	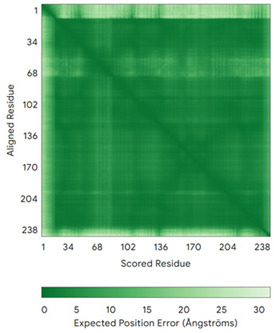
APE1 (P27695)	C65-C93 Docked Cys: C93 MPKRGKKGAVAEDGDELRTEPEAKKSKTAAKKNDKEAAGEGPALYEDPPDQKTSPSGKPATLKICSWNVDGLRAWIKKKGLDWVKEEAPDILCLQETKCSENKLPAELQELPGLSHQYWSAPSDKEGYSGVGLLSRQCPLKVSYGIGDEEHDQEGRVIVAEFDSFVLVTAYVPNAGRGLVRLEYRQRWDEAFRKFLKGLASRKPLVLCGDLNVAHEEIDLRNPKGNKKNAGFTPQERQGFGELLQAVPLADSFRHLYPNTPYAYTFWTYMMNARSKNVGWRLDYFLLSHSLLPALCDSKIRSKALGSDHCPITLYLAL	Code: 1E9N Technique: X-ray crystallography (Resol. 2.20 Å) Chain: A Residues: 2–318	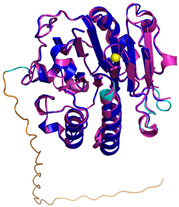 RMSD: 0.29 Å (256 C^α^ aligned)	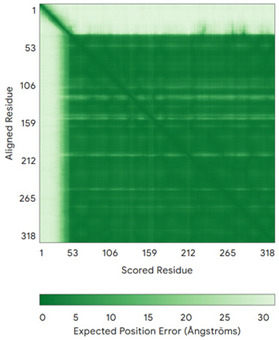
CHCHD2 (Q9Y6H1)	C114-C144, C124-C134 Docked Cys: C144 MPRGSRSRTSRMAPPASRAPQMRAAPRPAPVAQPPAAAPPSAVGSSAAAPRQPGLMAQMATTAAGVAVGSAVGHTLGHAITGGFSGGSNAEPARPDITYQEPQGTQPAQQQQPCLYEIKQFLECAQNQGDIKLCEGFNEVLKQCRLANGLA	-	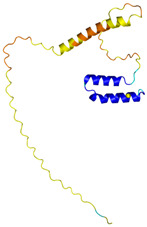	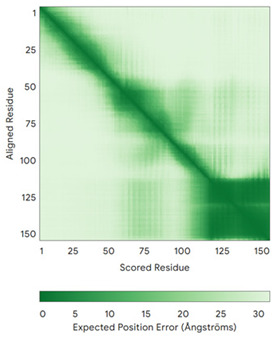
CHCHD3 (Q9NX63)	C183-C214, C193-C204 Docked Cys: C193 MGGTTSTRRVTFEADENENITVVKGIRLSENVIDRMKESSPSGSKSQRYSGAYGASVSDEELKRRVAEELALEQAKKESEDQKRLKQAKELDRERAAANEQLTRAILRERICSEEERAKAKHLARQLEEKDRVLKKQDAFYKEQLARLEERSSEFYRVTTEQYQKAAEEVEAKFKRYESHPVCADLQAKILQCYRENTHQTLKCSALATQYMHCVNHAKQSMLEKGG	-	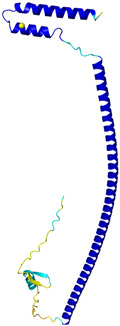	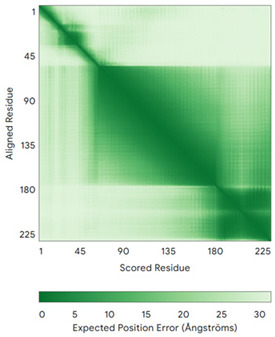
CMC1 (Q7Z7K0)	C31-C63, C41-C53 Docked Cys: C31 MALDPADQHLRHVEKDVLIPKIMREKAKERCSEQVQDFTKCCKNSGVLMVVKCRKENSALKECLTAYYNDPAFYEECKMEYLKEREEFRKTGIPTKKRLQKLPTSM	-	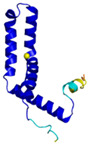	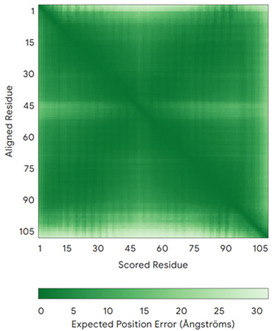
COX19 (Q49B96)	C30-C61, C40-C51 Docked Cys: C51 MSTAMNFGTKSFQPRPPDKGSFPLDHLGECKSFKEKFMKCLHNNNFENALCRKESKEYLECRMERKLMLQEPLEKLGFGDLTSGKSEAKK	-	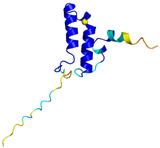	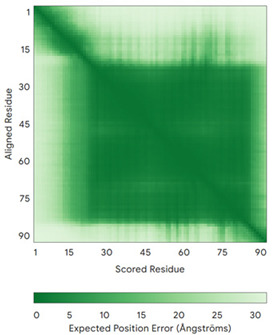
MICU1 (Q9BPX6)	- Docked Cys: C463 MFRLNSLSALAELAVGSRWYHGGSQPIQIRRRLMMVAFLGASAVTASTGLLWKRAHAESPPCVDNLKSDIGDKGKNKDEGDVCNHEKKTADLAPHPEEKKKKRSGFRDRKVMEYENRIRAYSTPDKIFRYFATLKVISEPGEAEVFMTPEDFVRSITPNEKQPEHLGLDQYIIKRFDGKKISQEREKFADEGSIFYTLGECGLISFSDYIFLTTVLSTPQRNFEIAFKMFDLNGDGEVDMEEFEQVQSIIRSQTSMGMRHRDRPTTGNTLKSGLCSALTTYFFGADLKGKLTIKNFLEFQRKLQHDVLKLEFERHDPVDGRITERQFGGMLLAYSGVQSKKLTAMQRQLKKHFKEGKGLTFQEVENFFTFLKNINDVDTALSFYHMAGASLDKVTMQQVARTVAKVELSDHVCDVVFALFDCDGNGELSNKEFVSIMKQRLMRGLEKPKDMGFTRLMQAMWKCAQETAWDFALPKQ	Code: 6LB7 Technique: X-ray crystallography (Resol. 2.10 Å) Chain: C Residues: 97–444	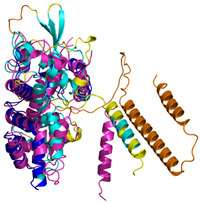 RMSD: 0.92 Å (270 C^α^ aligned)	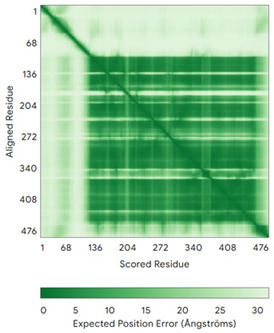
NDUFB10 (O96000)	C71-C78, C107-C119 Docked Cys: C107 MPDSWDKDVYPEPPRRTPVQPNPIVYMMKAFDLIVDRPVTLVREFIERQHAKNRYYYYHRQYRRVPDITECKEEDIMCMYEAEMQWKRDYKVDQEIINIMQDRLKACQQREGQNYQQNCIKEVEQFTQVAKAYQDRYQDLGAYSSARKCLAKQRQRMLQERKAAKEAAAATS	Code: 5XTD Technique: EM (Resol. 3.70 Å) Chain: d Residues: 1–171	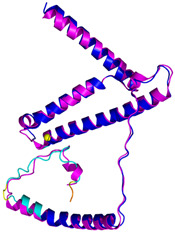 RMSD: 0.87 Å (156 C^α^ aligned)	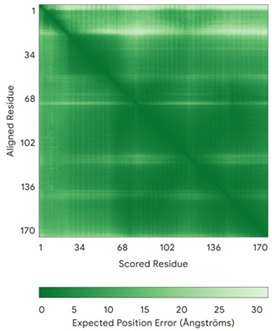
**Group IIb**				
Anamorsin (Q6FI81)	- MADFGISAGQFVAVVWDKSSPVEALKGLVDKLQALTGNEGRVSVENIKQLLQSAHKESSFDIILSGLVPGSTTLHSAEILAEIARILRPGGCLFLKEPVETAVDNNSKVKTASKLCSALTLSGLVEVKELQREPLTPEEVQSVREHLGHESDNLLFVQITGKKPNFEVGSSRQLKLSITKKSSPSVKPAVDPAAAKLWTLSANDMEDDSMDLIDSDELLDPEDLKKPDPASLRAASCGEGKKRKACKNCTCGLAEELEKEKSREQMSSQPKSACGNCYLGDAFRCASCPYLGMPAFKPGEKVLLSDSNLHDA	Code: 4M7R Technique: X-ray crystallography (Resol. 1.80 Å) Chain: A Residues: 1–172	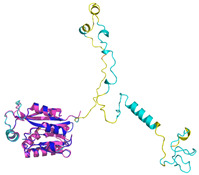 RMSD: 0.50 Å (150 C^α^ aligned)	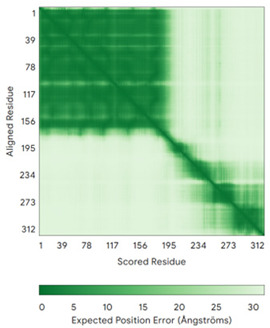
C9orf72 (Q96LT7)	- MSTLCPPPSPAVAKTEIALSGKSPLLAATFAYWDNILGPRVRHIWAPKTEQVLLSDGEITFLANHTLNGEILRNAESGAIDVKFFVLSEKGVIIVSLIFDGNWNGDRSTYGLSIILPQTELSFYLPLHRVCVDRLTHIIRKGRIWMHKERQENVQKIILEGTERMEDQGQSIIPMLTGEVIPVMELLSSMKSHSVPEEIDIADTVLNDDDIGDSCHEGFLLNAISSHLQTCGCSVVVGSSAEKVNKIVRTLCLFLTPAERKCSRLCEAESSFKYESGLFVQGLLKDSTGSFVLPFRQVMYAPYPTTHIDVDVNTVKQMPPCHEHIYNQRRYMRSELTAFWRATSEEDMAQDTIIYTDESFTPDLNIFQDVLHRDTLVKAFLDQVFQLKPGLSLRSTFLAQFLLVLHRKALTLIKYIEDDTQKGKKPFKSLRNLKIDLDLTAEGDLNIIMALAEKIKPGLHSFIFGRPFYTSVQERDVLMTF	Code: 6LT0 Technique: EM (Resol. 3.20 Å) Chain: C Residues: 1–481	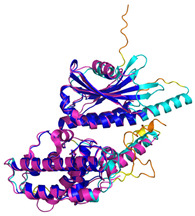 RMSD: 0.95 Å (364 C^α^ aligned)	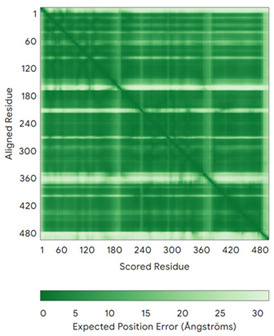
CHCHD1 (Q96BP2)	C45-C76, C55-C66 MATPSLRGRLARFGNPRKPVLKPNKPLILANRVGERRREKGEATCITEMSVMMACWKQNEFRDDACRKEIQGFLDCAARAQEARKMRSIQETLGESGSLLPNKLNKLLQRFPNKPYLS	Code: 6LT0 Technique: EM (Resol. 2.21 Å) Chain: A2 Residues: 1–118	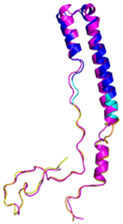 RMSD: 1.3 Å (110 C^α^ aligned)	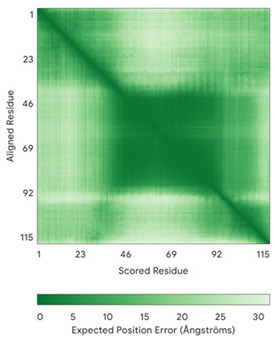
CHCHD5 (Q9BSY4)	C12-C44, C22-C34, C58-C89, C68-C79 MQAALEVTARYCGRELEQYGQCVAAKPESWQRDCHYLKMSIAQCTSSHPIIRQIRQACAQPFEAFEECLRQNEAAVGNCAEHMRRFLQCAEQVQPPRSPATVEAQPLPAS	Code: 2LQL Technique: NMR Chain: A Residues: 1–110	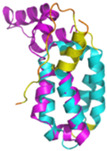 Residues 11–47 RMSD: 1.6 Å (35 C^α^ aligned) 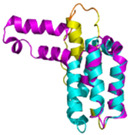 Residues 50–90 RMSD: 1.6 Å (36 C^α^ aligned)	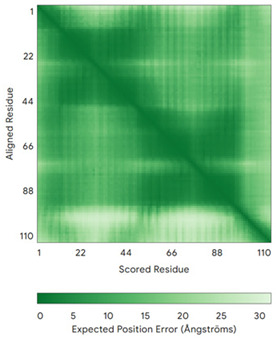
CHCHD7 (Q9BUK0)	C16-C47, C26-C37 MPSVTQRLRDPDINPCLSESDASTRCLDENNYDRERCSTYFLRYKNCRRFWNSIVMQRRKNGVKPFMPTAAERDEILRAVGNMPY	Code: 2LQT Technique: NMR Chain: A Residues: 1–85	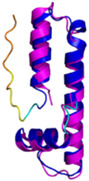 RMSD: 1.7 Å (69 C^α^ aligned)	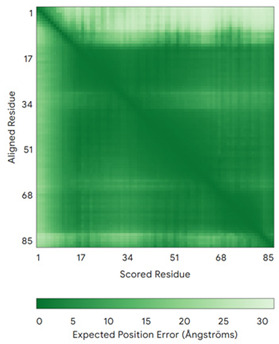
CHCHD10 (Q8WYQ3)	C102-C132, C112-C122 MPRGSRSAASRPASRPAAPSAHPPAHPPPSAAAPAPAPSGQPGLMAQMATTAAGVAVGSAVGHVMGSALTGAFSGGSSEPSQPAVQQAPTPAAPQPLQMGPCAYEIRQFLDCSTTQSDLSLCEGFSEALKQCKYYHGLSSLP	-	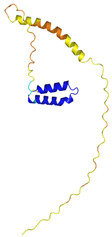	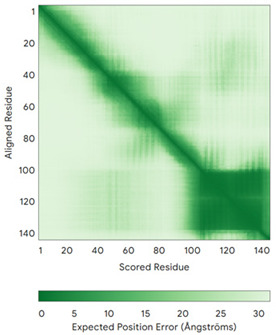
CMC2 (Q9NRP2)	C14-C47, C24-C37 MHPDLSPHLHTEECNVLINLLKECHKNHNILKFFGYCNDVDRELRKCLKNEYVENRTKSREHGIAMRKKLFNPPEESEK	-	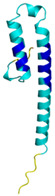	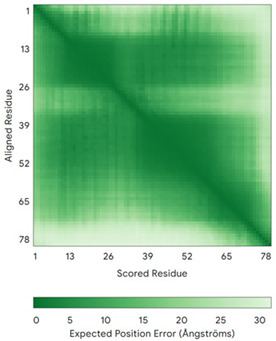
CMC4 (P56277)	C7-C38, C17-C28, C39-C50 MPQKDPCQKQACEIQKCLQANSYMESKCQAVIQELRKCCAQYPKGRSVVCSGFEKEEEENLTRKSASK	Code: 2HP8 Technique: NMR Chain: A Residues: 1–68	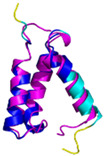 RMSD: 1.6 Å (60 C^α^ aligned)	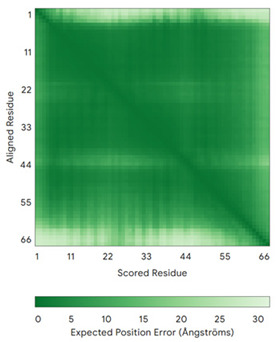
COA4 (Q9NYJ1)	C34-C64, C44-C54 MSTSVPQGHTWTQRVKKDDEEEDPLDQLISRSGCAASHFAVQECMAQHQDWRQCQPQVQAFKDCMSEQQARRQEELQRRQEQAGAHH	-	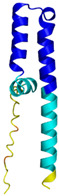	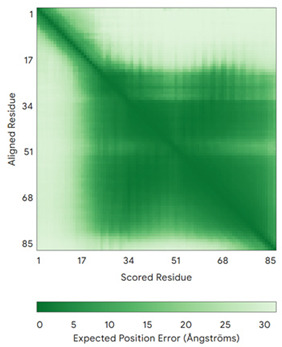
COA5 (Q86WW8)	- MPKYYEDKPQGGACAGLKEDLGACLLQSDCVVQEGKSPRQCLKEGYCNSLKYAFFECKRSVLDNRARFRGRKGY	-	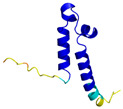	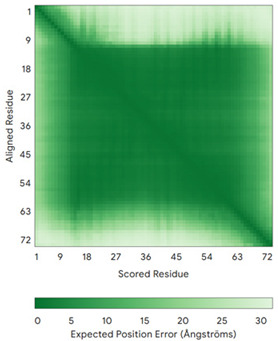
COA6 (Q5JTJ3)	C58-C90, C68-C79 MGPGGPLLSPSRGFLLCKTGWHSNRLLGDCGPHTPVSTALSFIAVGMAAPSMKERQVCWGARDEYWKCLDENLEDASQCKKLRSSFESSCPQQWIKYFDKRRDYLKFKEKFEAGQFEPSETTAKS	Code: 6PCE Technique: X-ray crystallography (Resol. 1.65 Å) Chain: A Residues: 50–119	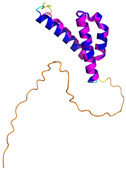 RMSD: 0.43 Å (50 C^α^ aligned)	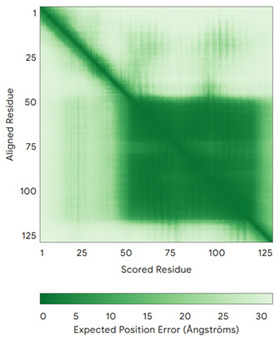
COA7 (Q96BR5)	C28-C37, C62-C71, C100-C111, C142-C150, C179-C187 MAGMVDFQDEEQVKSFLENMEVECNYHCYHEKDPDGCYRLVDYLEGIRKNFDEAAKVLKFNCEENQHSDSCYKLGAYYVTGKGGLTQDLKAAARCFLMACEKPGKKSIAACHNVGLLAHDGQVNEDGQPDLGKARDYYTRACDGGYTSSCFNLSAMFLQGAPGFPKDMDLACKYSMKACDLGHIWACANASRMYKLGDGVDKDEAKAEVLKNRAQQLHKEQQKGVQPLTFG	Code: 7MQZ Technique: X-ray crystallography (Resol. 2.39 Å) Chain: A Residues: 1–231	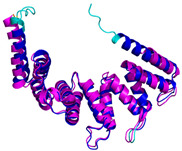 RMSD: 1.4 Å (202 C^α^ aligned)	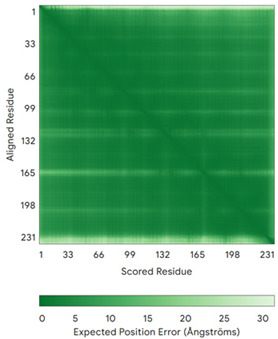
COX6B1 (P14854)	C30-C65, C40-C54 MAEDMETKIKNYKTAPFDSRFPNQNQTRNCWQNYLDFHRCQKAMTAKGGDISVCEWYQRVYQSLCPTSWVTDWDEQRAEGTFPGKI	Code: 5Z62 Technique: EM (Resol. 3.60 Å) Chain: H Residues: 5–86	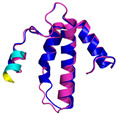 RMSD: 0.84 Å (73 C^α^ aligned)	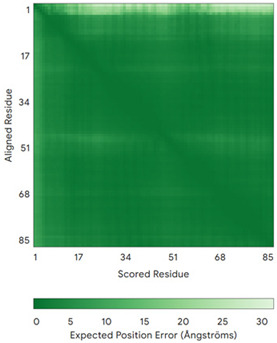
NDUFA8 (P51970)	C36-C66, C46-C56, C78-C110, C88-C100 MPGIVELPTLEELKVDEVKISSAVLKAAAHHYGAQCDKPNKEFMLCRWEEKDPRRCLEEGKLVNKCALDFFRQIKRHCAEPFTEYWTCIDYTGQQLFRHCRKQQAKFDECVLDKLGWVRPDLGELSKVTKVKTDRPLPENPYHSRPRPDPSPEIEGDLQPATHGSRFYFWTK	Code: 5XTD Technique: EM (Resol. 3.70 Å) Chain: u Residues: 4–172	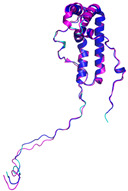 RMSD: 0.71 Å (149 C^α^ aligned)	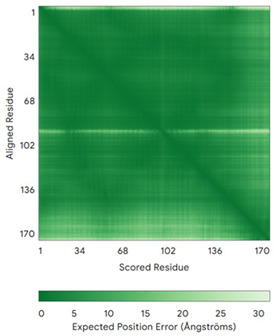
NDUFB7 (P17568)	C59-C90, C69-C80 MGAHLVRRYLGDASVEPDPLQMPTFPPDYGFPERKEREMVATQQEMMDAQLRLQLRDYCAHHLIRLLKCKRDSFPNFLACKQERHDWDYCEHRDYVMRMKEFERERRLLQRKKRREKKAAELAKGQGPGEVDPKVAL	Code: 5XTD Technique: EM (Resol. 3.70 Å) Chain: v Residues: 1–137	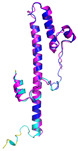 RMSD: 0.84 Å (96 C^α^ aligned)	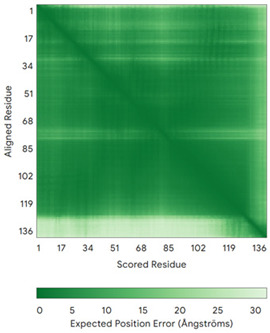
NDUFS5 (O43920)	C33-C66, C43-C56 MPFLDIQKRFGLNIDRWLTIQSGEQPYKMAGRCHAFEKEWIECAHGIGYTRAEKECKIEYDDFVECLLRQKTMRRAGTIRKQRDKLIKEGKYTPPPHHIGKGEPRP	Code: 5XTD Technique: EM (Resol. 3.70 Å) Chain: h Residues: 2–105	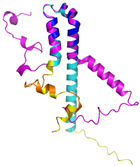 RMSD: 0.61 Å (34 C^α^ aligned)	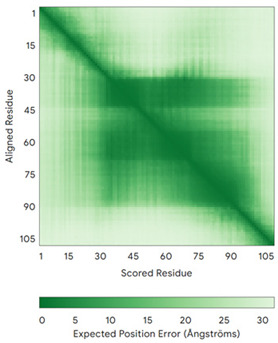
NDUFS8 (O00217)	- MRCLTTPMLLRALAQAARAGPPGGRSLHSSAVAATYKYVNMQDPEMDMKSVTDRAARTLLWTELFRGLGMTLSYLFREPATINYPFEKGPLSPRFRGEHALRRYPSGEERCIACKLCEAICPAQAITIEAEPRADGSRRTTRYDIDMTKCIYCGFCQEACPVDAIVEGPNFEFSTETHEELLYNKEKLLNNGDKWEAEIAANIQADYLYR	Code: 5XTD Technique: EM (Resol. 3.70 Å) Chain: B Residues: 35–210	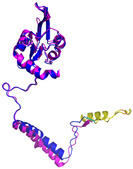 RMSD: 1.3 Å (155 C^α^ aligned)	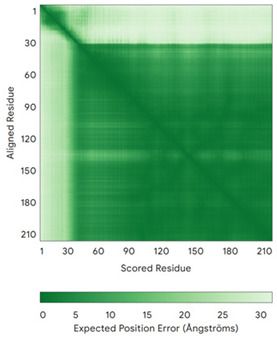
p53 (P04637)	- MEEPQSDPSVEPPLSQETFSDLWKLLPENNVLSPLPSQAMDDLMLSPDDIEQWFTEDPGPDEAPRMPEAAPPVAPAPAAPTPAAPAPAPSWPLSSSVPSQKTYQGSYGFRLGFLHSGTAKSVTCTYSPALNKMFCQLAKTCPVQLWVDSTPPPGTRVRAMAIYKQSQHMTEVVRRCPHHERCSDSDGLAPPQHLIRVEGNLRVEYLDDRNTFRHSVVVPYEPPEVGSDCTTIHYNYMCNSSCMGGMNRRPILTIITLEDSSGNLLGRNSFEVRVCACPGRDRRTEEENLRKKGEPHHELPPGSTKRALPNNTSSSPQPKKKPLDGEYFTLQIRGRERFEMFRELNEALELKDAQAGKEPGGSRAHSSHLKSKKGQSTSRHKKLMFKTEGPDSD	Code: 7XZZ Technique: EM (Resol. 4.07 Å) Chain: M Residues: 1–393	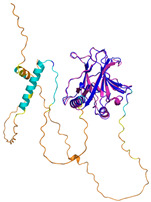 RMSD: 0.28 Å (180 C^α^ aligned)	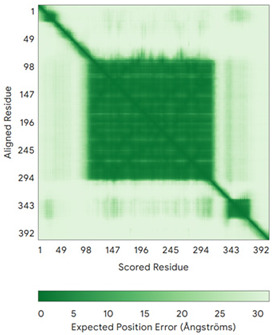
PINK1 (Q9BXM7)	- MAVRQALGRGLQLGRALLLRFTGKPGRAYGLGRPGPAAGCVRGERPGWAAGPGAEPRRVGLGLPNRLRFFRQSVAGLAARLQRQFVVRAWGCAGPCGRAVFLAFGLGLGLIEEKQAESRRAVSACQEIQAIFTQKSKPGPDPLDTRRLQGFRLEEYLIGQSIGKGCSAAVYEATMPTLPQNLEVTKSTGLLPGRGPGTSAPGEGQERAPGAPAFPLAIKMMWNISAGSSSEAILNTMSQELVPASRVALAGEYGAVTYRKSKRGPKQLAPHPNIIRVLRAFTSSVPLLPGALVDYPDVLPSRLHPEGLGHGRTLFLVMKNYPCTLRQYLCVNTPSPRLAAMMLLQLLEGVDHLVQQGIAHRDLKSDNILVELDPDGCPWLVIADFGCCLADESIGLQLPFSSWYVDRGGNGCLMAPEVSTARPGPRAVIDYSKADAWAVGAIAYEIFGLVNPFYGQGKAHLESRSYQEAQLPALPESVPPDVRQLVRALLQREASKRPSARVAANVLHLSLWGEHILALKNLKLDKMVGWLLQQSAATLLANRLTEKCCVETKMKMLFLANLECETLCQAALLLCSWRAAL	Code: 9EII Technique: EM (Resol. 2.75 Å) Chain: B Residues: 1–581	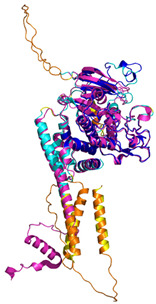 RMSD: 1.3 Å (401 C^α^ aligned)	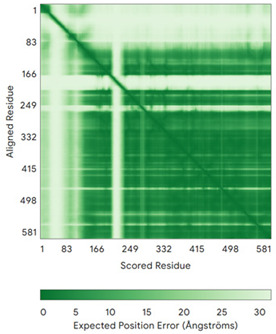
TIMM8A (O60220)	C43-C66, C47-C62 MDSSSSSSAAGLGAVDPQLQHFIEVETQKQRFQQLVHQMTELCWEKCMDKPGPKLDSRAEACFVNCVERFIDTSQFILNRLEQTQKSKPVFSESLSD	-	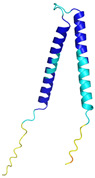	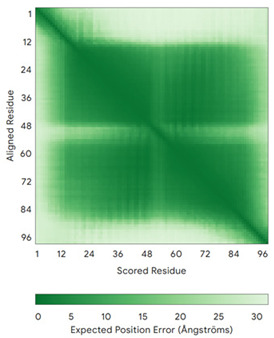
TIMM13 (Q9Y5L4)	C46-C69, C50-C65 MEGGFGSDFGGSGSGKLDPGLIMEQVKVQIAVANAQELLQRMTDKCFRKCIGKPGGSLDNSEQKCIAMCMDRYMDAWNTVSRAYNSRLQRERANM	-	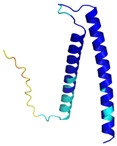	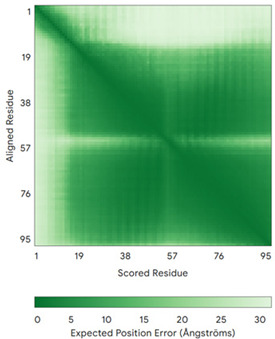
TRIAP1 (O43715)	C8-C47, C18-C37 MNSVGEACTDMKREYDQCFNRWFAEKFLKGDSSGDPCTDLFKRYQQCVQKAIKEKEIPIEGLEFMGHGKEKPENSS	Code: 6I3V Technique: X-ray crystallography (Resol. 1.98 Å) Chain: A Residues: 1–67	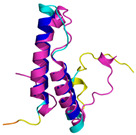 RMSD: 1.7 Å (49 C^α^ aligned)	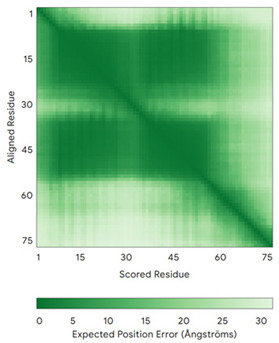

* When more PDB structures are available, the one with at highest resolution and/or covering the largest portion of the protein sequence has been reported.

### 6.1. The TIMM Protein Family

**Figure 3:**
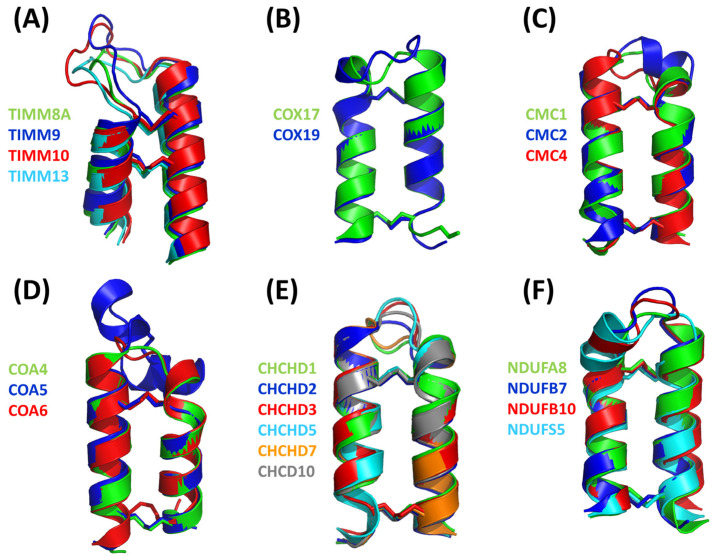
Structural superimposition of the helix–coil–helix motif in the AF predicted structures of selected members of the following protein families: TIMM (**A**), COX (**B**), CMC (**C**), COA (**D**), CHCHD (**E**), and NDUF_NADH (**F**). The disulfide bonds are shown as sticks.

Four different members (TIMM8A, TIMM9, TIMM10, and TIMM13) have been validated as hCHCHD4 substrates in this protein family. As reported in Table 4, the structure of two of them (TIMM9 and TIMM10) has been experimentally elucidated via X-ray diffraction analysis (PDB code 2BSK). In this crystallographic structure, the two TIMM proteins are involved in a hetero-hexameric assembly that exhibits an α-propeller topology [201]. The tertiary structure of the individual chains of TIMM9 and TIMM10 presents two helices with the Cys residues docked by hCHCHD4 located in the first one. In the crystal structure, these residues are in their oxidized state and are involved in the formation of disulfide bridges. As shown in Table 4, the AF3 predictions of the individual chains of TIMM9 and TIMM10 closely resemble those detected in the crystallographic structure (RMSD values < 1.0 Å). In these computed models, the reactive Cys are also in their oxidized state. Using AF3, we extended the structural information available for this family by predicting the structures of TIMM8A and TIMM13, which present similar secondary and tertiary structures (Table 4), despite the very limited sequence similarity. A global superimposition of the structures of all members of the family highlights this close structural similarity (Figure 3A). Interestingly, the docked Cys residues that are known only for TIMM9 (Cys28) and TIMM10 (Cys29) perfectly align with Cys42 and Cys46 of TIMM8A and TIMM13, respectively. This correspondence strongly suggests that these Cys residues of TIMM8A and TIMM13 are involved in the transient disulfide formation with hCHCHD4.

### 6.2. The COX Protein Family

Three distinct proteins (COX6B1, COX17, and COX19) are confirmed substrates of hCHCHD4 within the COX family. The structures of two of these proteins, COX6B1 and COX17, have been experimentally determined using cryo-EM (PDB code 5Z62) [202] and NMR (PDB code 2RNB) studies, respectively (Table 4) [203]. In the cryo-EM structure, COX6B1 is integrated into the giant complex formed by the 14-subunit-cytochrome c oxidase (complex IV), and its structure is characterized by a four-α-helices motif. All Cys residues are in their oxidized states (Cys30-Cys65 and Cys40-Cys54). The structure of COX17, whose reactive fragment was also studied in complex with hCHCHD4 (see Section 5), is characterized by the presence of two α-helices. In this model, four Cys residues, including the reactive Cys45, are involved in disulfide bonds (Cys26-Cys55 and Cys36-Cys45). The two other Cys residues (Cys23 and Cys24), which form a copper-binding motif, are in their reduced state. Comparison of these structures with the models predicted by AF3 reveals a close similarity, with RMSD values < 1.0 Å (Table 4). The predicted structure of COX19, which has not been experimentally characterized, closely resembles the experimental structure of COX17 (RMSD of the folded region = 0.41 Å) (Figure 3B), despite the limited sequence similarity. The structure similarity includes the reactive Cys residues. Attempts to overlap the structure of COX6B1 with that of either COX17 or COX19 do not provide meaningful results, thus confirming the distinctive structural properties of this protein.

### 6.3. The CMC Protein Family

In this family, three different proteins (CMC1, CMC2, and CMC4) have been validated as hCHCHD4 substrates (see Section 4). Among them, only CMC4 has been structurally characterized by solution NMR (PDB code 2HP8) [204]. This structure revealed a motif consisting of three α-helices that overlap well with those present in the AF3 model (RMSD of 1.6 Å) (Table 4). In these structures, six Cys residues are involved in disulfide bridges (Cys7-Cys38, Cys17-Cys28, and Cys39-Cys50), whereas Cys12 is in its reduced state.

**Figure 4:**
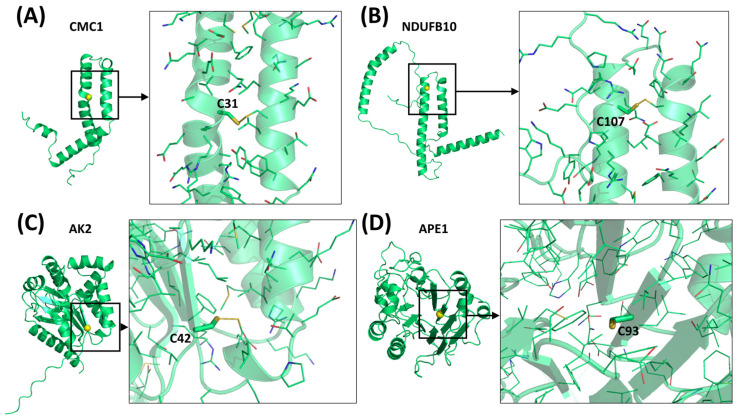
Representative examples of (partially) buried docked Cys residues in the AF3 models of hCHCHD4 substrates: CMC1 (**A**), NDUFB10 (**B**), AK2 (**C**), and APE1 (**D**). Cys residues are shown as yellow spheres in the overall models and as sticks in the zoomed-in views.

The prediction of the structures of CMC1 and CMC2 shows that they are both α-helical proteins. Although they carry some divergent regions, they show a well-conserved alpha–coil–alpha motif (Figure 3C). The structure analogy also extends to disulfide bridges, which include the reactive Cys residue (Cys31) of CMC1, which tethers the two helices (Figure 4A). Based on this overlay, by analogy, Cys14 of CMC2 is likely docked by hCHCHD4. Interestingly, the alpha–coil–alpha motif is also shared by CMC4 (Figure 3C). Considering the superimposition of CMC4 with CMC1, Cys7 of the former is structurally aligned with the reactive Cys31 of the latter, thus indicating its possible involvement in the disulfide bridge formation with hCHCHD4.

### 6.4. The CoA Protein Family

Within the COA family, four different proteins (COA4, COA5, COA6, and COA7) have been confirmed as substrates of hCHCHD4. Only two of them, COA6 (PDB code 6PCE) [205] and COA7 (PDB code 7MQZ) [155], have been structurally characterized by X-ray crystallography (Table 4). Although these proteins adopt all helical structures, they present quite different overall organization that is more intricate for COA7 (Figure 1). Indeed, the inspection of the COA6 tertiary structure reveals a three-helical bundle fold [205], with the first two helices linked by two disulfide bridges (Cys58-Cys90 and Cys68-Cys79). Two other Cys (Cys17 and Cys30) are located in the disordered N-terminal region of the protein. On the other hand, a banana-shaped fold composed of five helix–turn–helix repeats is exhibited by the crystal structure of COA7, with all Cys residues located in a buried environment [155]. The overall fold of the protein is stabilized by featuring five disulfide bonds, which tether the repeats (Table 4). This Cys-rich protein contains three additional Cys residues that are in their reduced state.

For both these structures, AF3 correctly predicts the structure of their folded regions (Table 4). For COA4 and COA5, which lack experimental structural data, AF3 predicts helical folds with helices connected by disulfide bridges. For both COA4 and COA5, covalent bonds tether the two helices, stabilizing the global structures of these proteins. As detected in the other members of the family, these proteins also present an all-helical structure. Pair-wise structural alignments provide reliable results for the superimposition of these proteins (Figure 3D), except for COA7, which presents a completely different fold (Figure 1). Notably, a satisfactory structural fitting of the disulfide bridges is observed.

### 6.5. The CHCHD Protein Family

Seven members of the CHCHD protein family (CHCHD1, CHCHD2, CHCHD3, CHCHD5, CHCHD7, and CHCHD10) have been confirmed as substrates of hCHCHD4. The inspection of the PDB shows that experimental structural data have been reported for CHCHD1, CHCHD5, and CHCHD7. CHCHD1 has been characterized as part of the large human mitoribosome complex through cryo-EM (PDB code 7QI4) [202]. The protein adopts a helical hairpin fold, with the two helices linked by two disulfide bonds (Cys45-Cys76 and Cys55-Cys66) (Figure 1). The structural characterization of CHCHD5 (PDB code 2LQL) and CHCHD7 (PDB code 2LQT) has been reported by a solution NMR study [110]. CHCHD5’s structure presents two weakly interacting coil–helix–coil–helix (CHCH) domains, which adopt a range of states because of hinge motions and hydrophobic interactions established by residues belonging to distinct domains. In addition to the CHCH domain, CHCHD7 has a third helix that forms hydrophobic interactions with one helix of the domain. Two disulfide bridges stabilize the CHCH domains of these two proteins.

AF3 predicts well the structures of CHCHD1 and CHCHD7 (Table 4). For CHCHD5, the prediction of the individual CHCH domains is excellent, while their mutual orientation is different from that reported in the NMR structure. This is not surprising since the experimental characterization of the protein has highlighted some domain motion in CHCHD5 [110].

The prediction of the three other members of the family (CHCHD2, CHCHD3, and CHCHD10) indicates that all share the CHCH domain, although variations on this common theme are observed (Figure 1). Interestingly, the structures of the CHCH domains of the six members of the family superimpose well (Figure 3E). Docked Cys residues are known for two proteins of the family (CHCHD2—Cys144 and CHCHD3—Cys193). Although their position in the model is not equivalent, they both participate in the stabilization of the helix–coil–helix motif.

### 6.6. The NDUF_NADH Dehydrogenase Protein Family

Within the NDUF_NADH family, five different proteins (NDUFA8, NDUFB7, NDUFB10, NDUFS5, and NDUFS8) have been confirmed as substrates of hCHCHD4. All these proteins that are subunits of the respiratory chain complex I have been found in the human Mitochondrial Respiratory Megacomplex I2III2IV2, which is structurally characterized by cryo-EM (PDB code 5XTD) [206]. The members of this family present exclusively α-helical secondary structures except for the NDUFS8, which presents a folded domain with an α-/β-structure. As shown in Figure 1, these proteins exhibit a marked structural diversity. In this family, only for the NDUFB10, the docked Cys (Cys107) has been identified. The inspection of the local structure of the protein indicates that in the oxidized state, this residue belongs to an α-helix, and it is involved in a disulfide bridge that stabilizes a helix–coil–helix motif (Figure 4B). Pair-wise structural alignments provide reliable results for the superimposition of all these proteins (Figure 3F), except for NDUFS8, which presents a completely different structural organization (Figure 1).

### 6.7. Other Substrates

For the substrates that are not grouped in a family, we integrated experimental data and prediction studies to obtain their three-dimensional organization, and we analyzed the local contexts of the reactive Cys residue if known. Here, we initially describe the substrate for which the docked Cys is known (Group IIa—AK2, APE1, and MICU1), and then we provide the available information for the global organization of the others (Anamorsin, C9orf72, p53, PINK1, and TRIAP1).

The structure of the protein AK2 has been deposited in the PDB (code 2C9Y), but no publication has been associated with it. The protein consists of three distinct domains, a large central one surrounded by two small domains, which are believed to undergo rearrangements in protein function. The overall organization of the domains reported in the experimental structure resembles that predicted by AF3 (Table 4). The inspection of the structure of the protein indicates that the reactive Cys42 is buried in the protein core (Figure 4C). This observation suggests that the occurrence of the reaction is not compatible with the fully folded state of the protein.

APE1 has been the subject of intensive structural characterizations. As shown in Figure 1, the protein adopts a α-/β-structure, with a disordered but functional N-terminal region [207]. As for the other substrates, the AF3 prediction is virtually identical to the experimental structure (Table 4). The inspection of the local environment of Cys93, the residue docked by hCHCHD4, indicates that it is heavily buried in the folded structure (Figure 4D), thus suggesting the redox reaction cannot occur with APE1 in its fully folded state.

The protein MICU1 has been structurally investigated by using X-crystallography and cryo-EM [208]. The folded region of the protein corresponds to the residues 97–444 (PDB code 6LB7) and does not include the docked Cys463 [208]. The AF3 model suggests that the region of the protein embodying this Cys has a partial helical character, although its pLDDT values do not enable very high confidence in the prediction (Figure 1).

For the other substrates, experimental structural characterizations were available for Anamorsin, C9orf72, p53, PINK1, and TRIAP1 (see Table 4). The AF3 predictions corroborate the experimental data without providing some extra meaningful information (Figure 1 and Table 3).

## 7. Conclusions and Perspectives

In the present work, we comprehensively analyzed the data available on the molecular and structural properties of the redox-regulated substrates of hCHCHD4. Since in modern biochemistry and biology, a full understanding of physio-pathological processes cannot arise from the acquisition of atomic-level information on the biomolecules involved, the structural perspective of this paper was strengthened by integrating the survey of the literature with atomic-level data extracted from databases containing either experimental or predicted three-dimensional structures of these substrates. In providing an updated view of the hCHCHD4 substrates that have been experimentally validated, our analyses highlight the notion that this protein can operate on a variety of structurally diversified substrates. Although in most cases, hCHCHD4 plays a crucial role in the formation of disulfide bridges that stabilize helix–coil–helix motifs of its substrates, significant variations on this common theme are observed, especially for substrates that have been more recently identified.

The integration of experimental structural data with predicted AlphaFold models provides a complete picture of the structural properties of these substrates. Indeed, we describe the predicted three-dimensional models for COX19, CHCHD2, CHCHD3, CHCHD10, CMC1, CMC2, COA4, COA5, PINK1, TIMM8A, and TIMM13, thus confirming the importance of machine learning approaches in current structural biology. As also demonstrated in other cases [209,210], these tools allow for shifting from single-protein structural analysis to comprehensive investigations of entire protein classes. The inspection of experimental and predicted three-dimensional models also provided, in some cases, interesting information on the putative site on the substrate specifically recognized by hCHCHD4 that could be validated in future investigations.

In contrast to the nearly complete structural data collected on the individual proteins involved in these partnerships, the scenario is completely different when the attention is focused on the three-dimensional models of the complexes formed by hCHCHD4 with substrates. Indeed, the structures of a few complexes formed by the protein with some canonical substrates, such as COX17 and TIMM9/TIMM10 proteins, are reported. This knowledge gap will likely be filled in the near future due to the methodological and technological advances of experimental structural biology techniques and the implementation of effective computational approaches that can predict the structure of protein complexes with remarkable reliability [25,26].

## Figures and Tables

**Table 1:** Literature survey protocol and outcome.

PUBMED Query	Total Papers
CHCHD4 substrates	25
CHCHD4 oxidoreductase	53
CHCHD4 redox	50
MIA40 substrates	89
MIA40 oxidoreductase	118
MIA40 redox	113

## Data Availability

Coordinates of the structures here predicted can be requested from the authors.
